# From ecDNA structure to tumor immune escape and therapeutic resistance: evidence, uncertainty, and clinical priorities

**DOI:** 10.3389/fonc.2026.1980333

**Published:** 2026-10-23

**Authors:** Shafee Ur Rehman

**Affiliations:** University of South Asia, Bishkek, Kyrgyzstan

**Keywords:** biomarker, extrachromosomal DNA, immune escape, precision oncology, therapeutic resistance

## Abstract

Extrachromosomal DNA (ecDNA) is a structurally distinct form of focal amplification that can increase oncogene dosage, reorganize enhancer–promoter interactions, and accelerate tumor evolution through unequal segregation. This review presents a single, evidence-based sequence: the structure of ecDNA confers an evolutionary capacity; selection then acts upon the genes and regulatory elements that are carried by ecDNA; the clones that have been selected may subsequently develop treatment-adaptive or immune-associated traits; and clinical application can only proceed once it has been demonstrated that ecDNA provides information that goes beyond what is seen in conventional amplification. The strongest evidence comes from unequal segregation, regulatory rewiring in specific models, reversible copy-number adaptation during targeted therapies, and replication-stress vulnerabilities in preclinical systems. In contrast, associations with reduced antigen presentation, altered interferon signaling, immune-cell exclusion, chemotherapy resistance, radiotherapy response, and immunotherapy failure remain context-dependent and not universally causal. When discussing the cGAS–STING pathway, intact nuclear ecDNA is differentiated from micronuclear DNA and damaged cytosolic fragments. Therapeutic strategies are classified based on direct preclinical evidence for ecDNA specificity, ecDNA-associated evidence, indirect biological reasoning, or a conceptual hypothesis; no strategy is presented as an established treatment directed at ecDNA. Detection methods are arranged from amplification screening to breakpoint-resolved inference to independent physical confirmation. Clinically useful driver biomarkers are separated from candidate ecDNA biomarkers that require prospective validation. The main conclusion is that ecDNA is an established driver of tumor evolution but has not yet been broadly validated as an immune-evasion mechanism, a predictive biomarker, or a routine therapeutic target.

## 1 Introduction

Amplification of focal oncogenes is common in aggressive cancers, but the amplified DNA can reside either within chromosomes or on extrachromosomal DNA (ecDNA) (1–3). Cancer-related ecDNA typically consists of large, chromatinized, and often circular DNA molecules that include oncogenes, genes involved in lineage survival, promoters, enhancers, and other regulatory elements (4–6). Unlike ordinary chromosomes, ecDNA generally lacks functional centromeres or telomeres and thus does not participate in normal chromosome-mediated segregation (7). Due to unequal inheritance, there is considerable variation from cell to cell in ecDNA copy number, which provides a basis for rapid clonal selection. Circularization can also place oncogenes near distant regulatory elements, and the open chromatin of ecDNA allows high levels of transcriptional activity (8, 9). In certain experimental models, ecDNA molecules form transcriptionally active hubs that enable cooperative interactions between enhancers and promoters (10). However, hub formation is not required for oncogene overexpression in all tumor models. Therefore, interpret ecDNA transcriptional output in light of tumor lineage, oncogene copy number, regulatory architecture, and experimental context.

Large-scale genomic studies have confirmed both how common ecDNA is and its prognostic significance. An analysis of 14,778 patients across 39 tumor types found that ecDNA was present in about 17.1% of tumor samples and was associated with treatment resistance and unfavorable clinical outcomes (11). The prevalence of ecDNA differed greatly among the various tumor types, which shows that the formation and maintenance of ecDNA are strongly influenced by lineage, oncogenic drivers, genomic-instability states, and selective pressures. Previous pan-cancer studies have also connected ecDNA with oncogene amplification, intratumor heterogeneity, aggressive disease, and worse survival (12–14). These findings offer strong evidence for the evolutionary importance of ecDNA but do not prove that the extrachromosomal state itself is responsible for all the phenotypes seen in tumors that are positive for ecDNA (**Table 1**).

**Table 1:** Prevalence of ecDNA across selected cancer types in a large pan-cancer analysis.

Cancer type or subgroup	ecDNA prevalence	Number of samples	Interpretation
Liposarcoma	54.9%	82	Highest reported prevalence in the analyzed cohort
Glioblastoma	49.1%	291	Frequent ecDNA involvement, often including oncogenic drivers
HER2-positive breast cancer	46.4%	Not separately reported in the summary	Indicates strong dependence on molecular subtype
Upper gastrointestinal adenocarcinoma	37.9%	Not separately reported in the summary	Relatively high prevalence in this disease group
Bladder cancer	24.6%	Not separately reported in the summary	Supports a role for ecDNA in selected urothelial cancers
Lung squamous cell carcinoma	22.4%	Not separately reported in the summary	More frequent than in several other lung cancer subtypes
Ovarian cancer	20.4%	Not separately reported in the summary	Demonstrates ecDNA involvement in a subset of ovarian tumors
Oligodendroglioma	0%	57	No ecDNA detected in this cohort
All analyzed tumor samples	17.1%	15,832 samples from 14,778 patients	The pooled estimate masks substantial lineage dependence

ecDNA, extrachromosomal DNA; HER2, human epidermal growth factor receptor 2.

Emerging studies have associated ecDNA positivity with reduced expression of antigen-presentation genes, altered inflammatory signaling, and diminished immune-cell infiltration (15–17). Nevertheless, most evidence linking ecDNA to immune escape is correlative. The observed immune phenotype may reflect the activity of amplified genes such as MYC, MYCN, EGFR, or MDM2; the chromosomal-instability processes that generated ecDNA; tumor-lineage characteristics; or differences in the surrounding microenvironment. Amplification of an immunoregulatory oncogene on ecDNA does not, by itself, demonstrate that ecDNA topology independently causes immune suppression. Establishing an ecDNA-specific effect requires experiments that compare extrachromosomal and chromosomal amplification while controlling for oncogene identity, copy number, and cellular background.

This critical narrative review follows the causal pathway from ecDNA structure and biological function to tumor evolution and clinical phenotype. It first defines ecDNA and examines its formation, regulatory architecture, transcriptional activity, and non-Mendelian inheritance, emphasizing the properties that generate intratumor heterogeneity and evolutionary capacity. It then critically evaluates whether immune escape and therapeutic resistance arise directly from the extrachromosomal state, from the biological activity of amplified oncogene cargo, or from associated chromosomal instability and microenvironmental factors. We then assess therapeutic opportunities based on supporting evidence, alongside a diagnostic hierarchy for ecDNA detection and a clear distinction between currently actionable oncogenic biomarkers and candidate ecDNA-based biomarkers requiring prospective validation. Throughout the review, we classify findings as direct ecDNA-specific evidence, ecDNA-associated evidence, oncogene-cargo or general cancer evidence, or mechanistic hypotheses.

## 2 Literature review approach

We conducted a structured narrative search of PubMed, Scopus, Web of Science, and Google Scholar for publications available through July 2026. Search concepts included “extrachromosomal DNA”, “ecDNA”, “double minutes”, “extrachromosomal circular DNA”, “oncogene amplification”, “immune escape”, “antigen presentation”, “cGAS–STING”, “treatment resistance”, “replication stress”, “CHK1”, “targeted therapy”, “detection”, and “precision oncology”, combined with Boolean operators as appropriate to each database. We screened reference lists of eligible articles to identify additional studies. We prioritized peer-reviewed original studies with structural confirmation of ecDNA, functional perturbation, longitudinal analysis, single-cell or spatial evidence, pan-cancer cohorts, or clinically annotated sequencing data. We used reviews for historical context and to cross-check findings. We included English-language full-text studies of human tumors, patient-derived samples, cancer cell lines, organoids, and relevant animal models; we included non-cancer circular-DNA studies only when they clarified terminology or methodology. We used conference abstracts, preprints, and trial registrations only to describe ongoing investigations and did not treat them as evidence of efficacy. We excluded studies when focal amplification was labeled ecDNA without structural or inferential support, when the reported phenotype could not be separated from general cancer biology, or when the article lacked sufficient methodological detail for attribution. Evidence was synthesized thematically and assigned to one of four categories: Level A, direct ecDNA-specific evidence with structural confirmation and functional or longitudinal support; Level B, ecDNA-associated evidence from validated models or cohorts without complete causal isolation; Level C, evidence derived mainly from amplified-oncogene biology, replication stress, chromosomal instability, or general cancer mechanisms; and Level D, conceptual or insufficiently tested hypotheses. Because this is a critical narrative review rather than a systematic review or meta-analysis, no protocol was registered, and no quantitative risk-of-bias or pooled-effect analysis was performed.

## 3 Biological characteristics of cancer-associated ecDNA

Extrachromosomal circular DNA encompasses a heterogeneous group of DNA molecules found outside conventional chromosomes. These molecules include microDNA, small polydisperse circles, telomeric circles, viral episomes, and large cancer-associated circular amplicons. The broader term eccDNA may refer to any extrachromosomal circular DNA species, including small circles detected in normal and malignant tissues. In this review, however, ecDNA refers specifically to large, chromatinized, usually circular DNA elements that carry amplified oncogenes or other cancer-associated regulatory sequences. These structures commonly range from several hundred kilobases to several megabases, although their size and composition vary considerably among tumors. Double minutes are small, paired, extrachromosomal chromatin bodies identified through cytogenetic examination. Modern sequencing and imaging studies have shown that many double minutes correspond to circular, structurally rearranged ecDNA molecules (18, 19). Nevertheless, ecDNA, eccDNA, and double minutes should not be used interchangeably. ecDNA is a molecular and structural category, whereas double minutes describe a cytogenetic appearance. Homogeneously staining regions represent intrachromosomal amplification and are therefore distinct from ecDNA, although ecDNA sequences may occasionally reintegrate into chromosomes and contribute to such structures.

### 3.1 Distinguishing features

Cancer-associated ecDNA possesses several structural and functional features that distinguish it from conventional chromosomes and intrachromosomal amplification. Most ecDNA molecules lack a functional centromere and frequently lack telomeres, although their precise topology and chromatin organization can vary (20–22). The absence of centromere-mediated segregation permits unequal distribution of ecDNA between daughter cells during mitosis. This non-Mendelian inheritance produces substantial cell-to-cell variation in ecDNA copy number and provides genetic diversity on which environmental or therapeutic selection can act. The resulting heterogeneity can facilitate rapid tumor adaptation, but it does not necessarily produce resistance or immune escape in every tumor. The phenotypic consequence depends on the genes and regulatory elements carried by ecDNA, their expression levels, and the selective conditions affecting the tumor. ecDNA molecules commonly contain complex rearrangements produced through chromosomal fragmentation, DNA breakage, abnormal replication, and error-prone repair. These rearrangements can juxtapose sequences that were widely separated in the original chromosome. Consequently, ecDNA may contain oncogenes together with promoters, distal enhancers, lineage-survival genes, transcription factors, noncoding RNAs, or immune-regulatory elements. Circularization can remove normal chromosomal boundaries and generate new enhancer–promoter relationships that increase transcription of ecDNA-encoded genes (23–25). These effects are directly attributable to ecDNA architecture when the circular structure, regulatory interaction, and transcriptional consequence have been experimentally demonstrated. In contrast, high expression of an amplified oncogene without structural confirmation cannot be assumed to result from ecDNA-specific enhancer rewiring.

The chromatin carried by ecDNA is frequently accessible and enriched for transcriptionally active regulatory features. This configuration can support oncogene expression that exceeds the level predicted from copy number alone. However, high transcription from ecDNA is not governed by a single universal mechanism. In some experimental systems, ecDNA molecules cluster into nuclear hubs that permit cooperative enhancer–promoter interactions between separate circles. Other studies have found that copy-number amplification primarily explains oncogene expression and does not require stable ecDNA clustering. Hub-dependent transcription should therefore be regarded as a context-dependent mechanism rather than an intrinsic feature of every ecDNA-positive cancer. The regulatory effects of ecDNA may extend beyond genes physically encoded on the circle. ecDNA-borne enhancers can regulate genes on the same molecule and, in selected experimental models, interact with regulatory regions located on other ecDNA molecules or conventional chromosomes. Similarly, ecDNA-encoded transcription factors and noncoding RNAs may alter broader cellular transcriptional programs (26–28). Evidence for these trans-regulatory effects remains more limited than evidence for oncogene dosage and local enhancer rewiring. Physical proximity alone does not prove functional regulation; causal attribution requires perturbing the ecDNA-associated regulatory element and measuring the proposed target response.

ecDNA can also undergo structural remodeling, changes in copy number, or reintegration into chromosomes during tumor evolution. These transitions complicate classification because the same amplified locus may exist as ecDNA in one cell population and as an intrachromosomal amplification in another. Accordingly, detection of focal amplification alone is insufficient to establish ecDNA. Reliable characterization should consider extrachromosomal localization, circular topology, breakpoint architecture, copy number, cellular heterogeneity, regulatory composition, chromatin accessibility, and transcriptional activity. This integrated approach is necessary to distinguish biological effects caused by ecDNA structure from those attributable to oncogene amplification or general genomic instability. The practical boundary established in this section is used throughout the review: an amplification becomes ecDNA evidence only when its extrachromosomal or circular architecture is supported, and a phenotype becomes ecDNA-specific only when topology contributes beyond gene identity and copy number (**Figure 1**).

**Figure 1:**
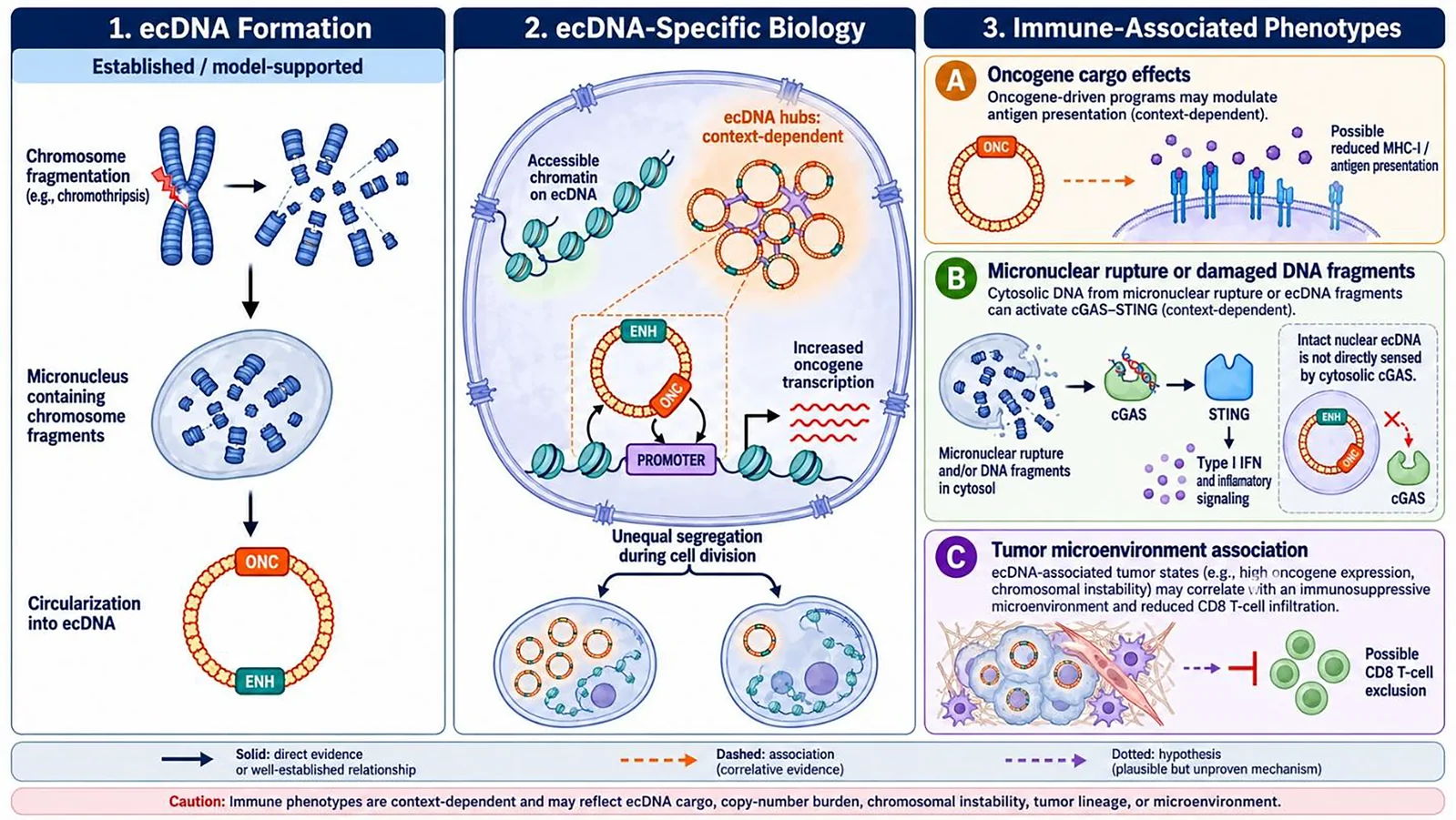
ecDNA formation, ecDNA-specific biology, and immune-associated phenotypes. Chromosomal fragmentation, including chromothripsis and micronuclear processing, can generate circular extrachromosomal DNA (ecDNA) containing oncogenes and regulatory elements. Within the nucleus, ecDNA may exhibit accessible chromatin, enhancer–promoter rewiring, context-dependent hub formation, increased oncogene transcription, and unequal segregation during cell division. Immune-associated phenotypes may arise through distinct mechanisms: oncogene-cargo effects on antigen presentation, cytosolic DNA released through micronuclear rupture or DNA damage, and tumor-microenvironmental changes associated with ecDNA-positive tumor states. Solid arrows indicate direct or well-supported relationships, dashed arrows indicate associations, and dotted arrows indicate plausible but unproven mechanisms. Immune phenotypes are context-dependent and may reflect ecDNA cargo, copy-number burden, chromosomal instability, tumor lineage, or the microenvironment. Schematic prepared specifically for this review; not drawn to scale.

## 4 Biogenesis of ecDNA

ecDNA can arise through several processes involving chromosome fragmentation, replication failure, structural rearrangement, and error-prone DNA repair (29, 30). These mechanisms are not mutually exclusive. A tumor may experience micronuclear fragmentation, breakage–fusion–bridge cycling, replication-fork collapse, and abnormal repair during successive stages of ecDNA formation and remodeling. The relative contribution of each process varies according to tumor lineage, genomic-instability state, DNA-repair capacity, oncogenic driver, and treatment exposure.

Distinguish the generation of a circular DNA molecule from its subsequent selection and amplification. Many DNA circles may be transient or confer no selective advantage. An ecDNA molecule becomes biologically important when it carries an oncogene, enhancer, lineage-survival gene, or another element that increases cellular fitness. Positive selection can then increase its copy number and promote further structural remodeling. Therefore, the prevalence of a particular ecDNA structure reflects both the mechanism that generated it and the selective advantage of its genetic and regulatory cargo (30).

### 4.1 Chromothripsis and micronuclei

Chromothripsis is a catastrophic process in which a chromosome or chromosomal region undergoes extensive fragmentation followed by incomplete or disordered reassembly (31, 32). Some fragments may rejoin into circular extrachromosomal structures rather than returning to their original chromosomal positions (33). This mechanism can explain the discontinuous genomic segments, complex breakpoint patterns, and co-amplification of oncogenes and regulatory elements observed in some ecDNA molecules. Micronuclei provide one cellular setting in which chromothripsis and ecDNA formation may occur. They arise when a chromosome or chromosome fragment fails to segregate normally and becomes enclosed within a separate nuclear membrane (34, 35). DNA replication and repair within micronuclei are frequently defective. Rupture of the micronuclear envelope can expose the enclosed DNA to damage, nucleases, and abnormal repair processes, producing fragmented DNA that may subsequently be circularized. However, micronuclear rupture should not be treated as a universal route of ecDNA formation. Not all micronuclear DNA forms ecDNA, and ecDNA may arise through mechanisms that do not involve detectable micronuclei. Furthermore, the presence of ecDNA and chromothripsis in the same tumor does not by itself establish that chromothripsis generated the ecDNA. Breakpoint reconstruction, temporal analysis, or experimental modeling is required to support a causal relationship. When a newly formed circle contains fitness-promoting cargo, positive selection may increase its abundance during tumor evolution (36).

### 4.2 Breakage–fusion–bridge cycles

Telomere loss or dysfunction can promote end-to-end chromosome fusion and dicentric chromosome formation. During mitosis, the two centromeres of a dicentric chromosome may be pulled toward opposite spindle poles, creating an anaphase bridge that eventually breaks. The resulting chromosome ends may fuse again during subsequent cell cycles, initiating repeated breakage–fusion–bridge cycles (37–39). These cycles generate inverted duplications, deletions, complex rearrangements, and progressive amplification of genomic regions. Amplified segments may subsequently be excised and circularized, providing a possible route to ecDNA formation (40). Conversely, ecDNA may reintegrate into a chromosome and contribute to intrachromosomal amplification. Breakage–fusion–bridge structures and ecDNA can therefore coexist or represent different stages of amplicon evolution. Copy-number patterns compatible with breakage–fusion–bridge cycling do not prove that an amplified region is extrachromosomal. Distinguishing these states requires structural reconstruction and, ideally, cytogenetic or other orthogonal confirmation. The contribution of breakage–fusion–bridge cycling to ecDNA formation appears to be tumor- and context-dependent rather than universal.

### 4.3 Replication-associated errors

Replication-fork stalling, fork collapse, unscheduled re-replication, abnormal origin firing, and template switching can generate duplicated, deleted, or rearranged genomic segments. Error-prone processing of these replication intermediates may join broken DNA ends and contribute to circular amplicon formation (41, 42). Replication-associated mechanisms are particularly plausible in tumors with oncogene-induced replication stress or defects in replication-fork protection.

Replication stress can act both before and after ecDNA formation. Initially, it may generate DNA breaks and rearrangements that contribute to circle formation. Once ecDNA is established, high copy number and intense transcription can increase the burden of DNA replication and create transcription–replication conflicts. These conflicts may cause additional fork stalling, DNA breakage, and structural remodeling. This continuing instability can diversify ecDNA structures during tumor progression. Nevertheless, replication stress is common in ecDNA-negative cancers and is not specific to ecDNA. Its presence alone cannot demonstrate an ecDNA-forming mechanism. Direct attribution requires breakpoint-resolved structural evidence or experimental observation showing that replication failure generated the circular molecule.

### 4.4 Double-strand break repair

DNA double-strand breaks can contribute to ecDNA formation when excised or fragmented genomic segments are joined through non-homologous end joining, microhomology-mediated end joining, or other repair processes. These pathways can reconnect DNA ends without extensive sequence homology and may produce deletions, insertions, short duplications, or abnormal junctions (42–44). Repair can circularize a single chromosomal segment or combine fragments derived from distant regions of the same chromosome or from different chromosomes.

Short microhomologies at some ecDNA breakpoint junctions support microhomology-mediated repair. However, breakpoint signatures vary substantially among tumors, and no single repair pathway explains all ecDNA structures (45). Some junctions are compatible with direct end joining, whereas others suggest replication-associated template switching or more complex combinations of damage and repair. Breakpoint sequence should therefore be interpreted as evidence consistent with a particular mechanism rather than definitive proof of that mechanism.

The DNA-repair pathway involved in initial circle formation may also differ from the pathways responsible for later ecDNA amplification and remodeling. Studies should distinguish these stages and avoid inferring the entire evolutionary history of an ecDNA molecule from one breakpoint pattern.

### 4.5 Viral–human hybrid ecDNA

In virus-associated cancers, viral integration and host-genome rearrangement can generate hybrid ecDNA containing both viral and human DNA. This phenomenon has been characterized particularly in human papillomavirus-associated cancers (46, 47). Viral integration can produce host-genome breakage, amplification, and circularization, placing the viral oncogenes *E6* and *E7* near viral or hijacked human regulatory elements. These hybrid molecules can support high viral-oncogene expression and may contribute to cell-cycle dysregulation and malignant growth. Their functional effect depends on both the viral oncogenes and the regulatory architecture created by circularization. Accordingly, the phenotype should not be attributed solely to ecDNA topology or solely to viral gene dosage. Hybrid viral–human ecDNA provides one of the more specific experimental settings for testing ecDNA-associated regulatory dependencies. CRISPR interference directed against hybrid-ecDNA enhancers has reduced viral-oncogene expression and tumor growth in experimental models (48–51). Inhibition of enhancer-associated transcriptional proteins has also shown activity in selected models. These findings constitute preclinical evidence in a defined HPV-associated context. They do not establish CRISPR interference or BET-family inhibition as broadly effective treatments for all ecDNA-positive cancers. Clinical efficacy, delivery, durability, selectivity, and applicability to nonviral ecDNA remain unresolved. Biogenesis therefore explains how a selectable circular amplicon can arise, but it does not by itself explain immune escape or resistance; those phenotypes emerge only when a particular ecDNA cargo is favored under a defined selective pressure.

## 5 ecDNA architecture and transcriptional regulation

The transcriptional activity of ecDNA reflects both increased gene dosage and the regulatory organization of the amplified molecule. Circularization can alter chromatin accessibility, remove normal chromosomal boundaries, and juxtapose oncogenes with regulatory elements that were previously separated. These changes may increase transcription beyond what copy number alone would predict (1, 12, 23). However, not every ecDNA molecule has the same regulatory architecture. Transcriptional output varies with oncogene cargo, enhancer composition, chromatin state, copy number, tumor lineage, and cellular context. Gene amplification should therefore be distinguished from regulatory effects specifically produced by the extrachromosomal structure.

### 5.1 Accessible chromatin

Although ecDNA is assembled into nucleosomes, many ecDNA molecules exhibit accessible chromatin, active histone modifications, and occupancy by transcription factors, coactivators, and RNA polymerase II (1, 20, 23). These features can support strong expression of ecDNA-encoded oncogenes. In some models, transcriptional output exceeds the level predicted from copy number, suggesting that chromatin accessibility and regulatory architecture contribute to expression. Nevertheless, accessible chromatin is neither unique to ecDNA nor uniform across all ecDNA-positive tumors. Highly active chromosomal amplicons can also display accessible chromatin, and some ecDNA regions may be transcriptionally inactive. Chromatin accessibility alone cannot therefore establish that an amplified locus is located on ecDNA. Demonstrating an ecDNA-specific regulatory effect requires structural confirmation of the ecDNA molecule and comparison with an appropriate chromosomal amplification or copy-number-matched control.

### 5.2 Enhancer hijacking

ecDNA formation can bring oncogenes into proximity with enhancers that were distant or insulated in the original chromosome. Circularization may also remove boundary elements that normally restrict regulatory interactions. The resulting enhancer–promoter relationships can sustain high oncogene transcription (3, 12). Preferential retention of functional enhancers during ecDNA evolution suggests that selection can operate on regulatory composition as well as oncogene content. Enhancer hijacking is attributable to ecDNA when a breakpoint-resolved circular structure places an oncogene beside a new enhancer and perturbing that enhancer changes oncogene expression. Without this evidence, high expression from an amplified locus may reflect copy number or conventional chromosomal regulation. Moreover, two ecDNA molecules carrying the same oncogene may differ in transcriptional output because they contain different enhancers, promoters, insulators, or structural junctions. The immunological consequences of enhancer hijacking remain incompletely defined. An ecDNA-associated enhancer could alter an immune-regulatory gene or intensify oncogene expression in ways that affect antigen presentation or cytokine signaling. Describe such findings as cargo- or enhancer-dependent unless experiments show that ecDNA topology itself produces the immune phenotype.

### 5.3 ecDNA hubs

Multiple ecDNA molecules can cluster within the nucleus and form structures described as ecDNA hubs. In selected experimental models, these hubs concentrate transcription factors and coactivators and permit cooperative enhancer–promoter interactions between separate ecDNA molecules (10). This organization may increase oncogene expression and allow structurally distinct ecDNA species to participate in a shared transcriptional program. The importance of hub formation depends on context. Other studies, particularly in glioblastoma stem-cell models, have reported that copy number mainly explains oncogene expression and that stable spatial clustering is not required (28). Physical proximity between ecDNA molecules also does not, by itself, demonstrate functional enhancer sharing. Hub-mediated transcription should therefore not be presented as a universal ecDNA characteristic. No hub-specific molecular component has yet been validated as a clinical therapeutic target. Proteins involved in transcriptional condensates, enhancer activity, or chromatin organization also regulate conventional chromosomes. Consequently, proposed disruption of ecDNA hubs remains an experimental concept requiring evidence of selectivity, target engagement, and therapeutic benefit in ecDNA-positive models compared with matched chromosomal-amplification controls.

### 5.4 Trans-regulatory effects

The regulatory influence of ecDNA may extend beyond genes physically located on the circle. Experimental studies suggest that ecDNA-borne enhancers can contact chromosomal loci and alter transcription of genes elsewhere in the genome (12). In addition, transcription factors or noncoding RNAs encoded on ecDNA may affect broader gene-expression networks. These observations support the possibility that ecDNA functions as a mobile regulatory platform. Evidence for trans-regulatory activity is less extensive than evidence for gene dosage and local enhancer rewiring. Three-dimensional contact between an ecDNA element and a chromosomal locus does not prove that the contact changes transcription. Causal attribution requires perturbing the ecDNA-associated enhancer or regulatory element, demonstrating a corresponding change in the proposed chromosomal target, and controlling for oncogene dosage and cellular state. The functional interpretation of ecDNA should therefore integrate its complete sequence content, breakpoint architecture, copy number, chromatin accessibility, enhancer activity, transcription-factor occupancy, and three-dimensional interactions. These data are needed to distinguish direct effects of ecDNA architecture from consequences of oncogene amplification or general transcriptional dysregulation (**Table 2**). Taken together, dosage is the most general transcriptional consequence, enhancer rewiring is directly supported in defined structures, hub-dependent cooperation is context-dependent, and genome-wide trans-regulation remains the least established mechanism.

**Table 2:** Molecular characteristics of ecDNA and the strength of their inferred biological and immune consequences.

ecDNA characteristic	Directly supported biological effect	Proposed immune relevance	Evidence attribution and limitation
Circular, acentric structure	Enables extrachromosomal persistence and unequal segregation	May generate subclones with different immune phenotypes	Unequal segregation is directly supported; immune consequences remain largely inferred
High oncogene copy number	Increases expression of amplified drivers and promotes clonal selection	Amplified drivers may alter interferon signaling, antigen presentation, or immune-cell recruitment	Primarily an oncogene-cargo effect rather than proof of an ecDNA-topology effect
Accessible chromatin	Supports binding of transcriptional machinery and high gene expression	May intensify oncogene-mediated immunoregulatory programs	Frequent but not universal; accessibility is not specific to ecDNA
Enhancer hijacking	Creates new enhancer–promoter relationships	May alter expression of immune-regulatory genes indirectly	Directly supported in selected ecDNA models; immune effects require separate validation
ecDNA hubs	Support cooperative transcription in selected models	Could intensify oncogene-driven immune suppression	Context-dependent; other models report copy-number-driven expression without hub dependence
Trans-regulatory activity	May influence genes located outside ecDNA	Could affect cytokine, chemokine, interferon, or antigen-presentation pathways	Emerging experimental evidence; physical contact alone does not establish regulation
Non-Mendelian segregation	Produces rapid cell-to-cell variation in ecDNA copy number	May permit selection of immune-resistant subclones	Evolutionary mechanism is supported; antigen-specific effects remain insufficiently characterized
Coordinated inheritance	Can preserve combinations of distinct ecDNA species	May maintain complementary growth and immune-regulatory programs	Demonstrated in selected models; clinical and immune significance remains uncertain
Structural plasticity	Permits rearrangement, copy-number change, and acquisition or loss of regulatory elements	May alter immunogenic or immune-regulatory cargo	Structural evolution is supported; specific immune effects are context-dependent
Chromosomal reintegration	Can convert an extrachromosomal amplicon into intrachromosomal amplification	May preserve an oncogenic phenotype after apparent ecDNA loss	Reintegration is biologically plausible and observed; immune consequences are unproven
Replication stress	Creates transcription–replication conflicts and DNA-damage-response dependencies	Damaged or micronuclear DNA may activate innate sensing	Direct preclinical evidence supports stress vulnerability; immune activation is not universally observed
Tumor-specific breakpoint junctions	Provide molecular identifiers of individual ecDNA structures	Junction-derived transcripts or peptides may be immunogenic	Useful for structural detection; immunogenicity, vaccination, and therapeutic editing remain investigational
Viral–human hybrid ecDNA	Sustains viral-oncogene expression through rearranged regulatory architecture	Viral antigens may interact with tumor immune selection	Direct evidence exists in selected HPV-associated models but should not be generalized to all ecDNA

## 6 Non-Mendelian inheritance and accelerated cancer evolution

Because ecDNA generally lacks a functional centromere, it does not follow the tightly regulated segregation pattern of conventional chromosomes. ecDNA molecules replicate during the cell cycle and can associate with mitotic chromosomes, but individual copies may be distributed unequally between daughter cells. Live-cell imaging and experimental studies have demonstrated that this non-Mendelian inheritance produces substantial cell-to-cell variation in ecDNA copy number and, consequently, in the dosage of ecDNA-encoded genes (46, 47). Unequal segregation generates heritable variation more rapidly than the gradual accumulation of new nucleotide-level mutations. It does not, however, determine which cellular phenotype will be selected. When high oncogene dosage increases proliferation or survival, cells carrying more ecDNA copies may expand. Under different conditions, high copy number may impose replication, transcriptional, or metabolic costs. Treatment directed against an ecDNA-encoded oncogene may also favor cells with fewer copies of the targeted ecDNA. The direction of selection therefore depends on oncogene cargo, copy-number burden, tumor lineage, microenvironmental conditions, and treatment exposure.

Anticancer therapy can alter the distribution of pre-existing ecDNA states within a tumor. In selected experimental models, treatment has favored cells that reduce or lose ecDNA carrying the targeted oncogene, whereas withdrawal of treatment has permitted the ecDNA-bearing population to re-emerge. This provides direct evidence that ecDNA copy-number plasticity can support reversible adaptation without requiring a new resistance mutation. Such behavior has not been demonstrated for every ecDNA-associated oncogene or treatment. Therefore, attribute resistance to ecDNA inheritance only when longitudinal or functional evidence shows that changes in ecDNA abundance or structure accompany the resistant phenotype. Although ecDNA segregation is often described as random, recent studies indicate that distinct ecDNA molecules may sometimes show coordinated inheritance. Physical association with mitotic chromosomes or co-segregation of separate ecDNA species may help maintain complementary oncogenic programs (52, 53). These observations do not invalidate non-Mendelian inheritance. Instead, they suggest that ecDNA segregation ranges from largely stochastic distribution to partially coordinated retention, depending on the molecular structure, cellular model, and mechanisms of chromosomal tethering. The prevalence and functional importance of coordinated inheritance in patient tumors remain uncertain. The evolutionary consequences of ecDNA segregation also depend on the fitness effects of its cargo. Unequal inheritance provides variation on which selection can act, while the tumor environment determines which ecDNA state is favored. This process can increase intratumor heterogeneity and accelerate adaptation, but it does not invariably produce treatment resistance, immune escape, recurrence, or poor outcome. Those clinical consequences require additional evidence linking a specific change in ecDNA copy number or structure to the observed phenotype. Thus, non-Mendelian inheritance is best understood as an ecDNA-specific mechanism that increases evolutionary potential rather than as a universal determinant of aggressive tumor behavior. This section supplies the evolutionary bridge for the remainder of the review: ecDNA-specific unequal inheritance creates selectable variation, whereas the cargo and environment determine whether that variation produces immune escape, resistance, or no measurable advantage.

## 7 ecDNA and tumor immune escape

The relationship between ecDNA and antitumor immunity remains incompletely understood. Transcriptomic and pan-cancer studies have associated ecDNA-positive tumors with reduced antigen-presentation programs, altered interferon signaling, and differences in immune-cell infiltration (50, 54). These observations support an association between ecDNA positivity and immune suppression, but they do not establish that ecDNA topology independently causes immune escape. At least four explanations may contribute to the immune phenotype of an ecDNA-positive tumor. First, ecDNA structural properties, including unequal segregation and regulatory rewiring, may influence cellular heterogeneity or gene expression. Second, oncogenes and immune-regulatory elements carried on ecDNA may alter antigen presentation, cytokine production, or immune-cell recruitment. Third, the chromosomal instability and micronuclear damage associated with ecDNA formation may affect innate DNA sensing. Fourth, tumor lineage, treatment exposure, stromal composition, and other microenvironmental factors may confound the observed association. These mechanisms are not mutually exclusive, and their relative importance probably differs among tumor types. Therefore, reserve the term “ecDNA-mediated immune escape” for findings supported by functional experiments that isolate the extrachromosomal contribution. Describe cohort-level associations as ecDNA-associated immune phenotypes, and identify effects driven by MYC, EGFR, MYCN, MDM2, or other amplified genes as oncogene-cargo-mediated mechanisms.

### 7.1 Reduced antigen presentation

Pan-cancer analyses have reported lower expression of genes involved in major histocompatibility complex class I and class II antigen presentation in some ecDNA-positive tumors (50, 54). Reduced major histocompatibility complex class I activity can limit the presentation of tumor-derived peptides to CD8-positive cytotoxic T cells. Reduced major histocompatibility complex class II activity may also affect CD4-positive T-cell responses and communication with antigen-presenting cells. These changes could contribute to impaired immune recognition. The available evidence is predominantly transcriptomic and associative. Reduced expression of antigen-presentation genes does not demonstrate that ecDNA directly suppresses antigen presentation at the protein or functional level. The phenotype may instead reflect amplified oncogene activity, tumor-lineage differences, epigenetic silencing, loss of interferon responsiveness, human leukocyte antigen alterations, or broader copy-number instability. Measurements derived from bulk tumor tissue may also be affected by differences in tumor purity and immune-cell abundance.

A causal ecDNA-specific mechanism would require more controlled evidence. Ideally, studies should compare cells carrying an oncogene on ecDNA with cells carrying a copy-number-matched intrachromosomal amplification in the same genetic background. Experimental gain, depletion, or structural conversion of ecDNA should then be linked to changes in major histocompatibility complex expression, peptide presentation, and T-cell recognition. Until such findings are reproduced across tumor types, reduced antigen presentation should be described as an ecDNA-associated phenotype rather than an intrinsic property of all ecDNA-positive cancers. The relationship may also be context-dependent. Some ecDNA-positive tumors may retain antigen-presentation activity, particularly when interferon signaling remains functional or when the amplified cargo does not suppress immune pathways. ecDNA status should therefore be interpreted alongside tumor lineage, oncogene identity, human leukocyte antigen status, interferon competence, treatment exposure, and the composition of the immune microenvironment.

### 7.2 cGAS–STING signaling

The cyclic GMP–AMP synthase–stimulator of interferon genes pathway detects double-stranded DNA that is abnormally present in the cytosol. Cytosolic DNA binding to cyclic GMP–AMP synthase promotes production of cyclic GMP–AMP, activation of stimulator of interferon genes, phosphorylation of TANK-binding kinase 1 and interferon regulatory factor 3, and induction of type I interferon and inflammatory signaling. The DNA species responsible for pathway activation in ecDNA-positive tumors must be defined carefully. Intact ecDNA is predominantly retained within the nucleus and should not be assumed to be routinely accessible to cytosolic cyclic GMP–AMP synthase. Cytosolic DNA may instead originate from ruptured micronuclei, damaged ecDNA-derived fragments, chromosome fragments, mitochondrial DNA, or other products of genomic instability. Consequently, evidence that an ecDNA-positive tumor activates or suppresses cyclic GMP–AMP synthase–stimulator of interferon genes signaling does not establish that intact ecDNA is the directly sensed ligand.

This distinction is particularly important because ecDNA-positive cancers often coexist with chromosomal instability and micronuclear damage. Micronuclear-envelope rupture can release DNA into the cytosol and activate innate sensing. Conversely, tumors experiencing persistent genomic instability may undergo negative selection for reduced cyclic GMP–AMP synthase, stimulator of interferon genes, or downstream interferon activity. Epigenetic silencing, pathway mutation, increased clearance of cytosolic DNA, impaired downstream signaling, or adaptation to chronic stimulation could therefore permit survival despite continued DNA damage (50). The biological consequences of pathway activation are also context-dependent. Acute activation of the cyclic GMP–AMP synthase–stimulator of interferon genes pathway can promote type I interferon production, dendritic-cell activation, antigen presentation, and cytotoxic T-cell recruitment. In contrast, persistent or low-level signaling may contribute to chronic inflammation, immune tolerance, tissue damage, or tumor progression. Differences in tumor lineage, pathway integrity, cellular compartment, signal duration, and treatment history may therefore explain apparently contradictory findings.

Pharmacological restoration of innate DNA sensing and the use of stimulator of interferon genes agonists represent investigational strategies rather than established ecDNA-directed treatments. Their evaluation should confirm the source of cytosolic DNA, demonstrate that the pathway remains functionally responsive, and compare ecDNA-positive and appropriately matched ecDNA-negative models. Potential combinations with radiotherapy or immune-checkpoint inhibition require careful assessment because pathway activation may produce either antitumor or protumor effects. As illustrated in **Figure 2**, intact nuclear ecDNA should be distinguished visually and mechanistically from micronuclear DNA, damaged ecDNA-derived fragments, and other cytosolic DNA species. This distinction prevents the unsupported implication that intact ecDNA is routinely detected directly by cytosolic cyclic GMP–AMP synthase.

**Figure 2:**
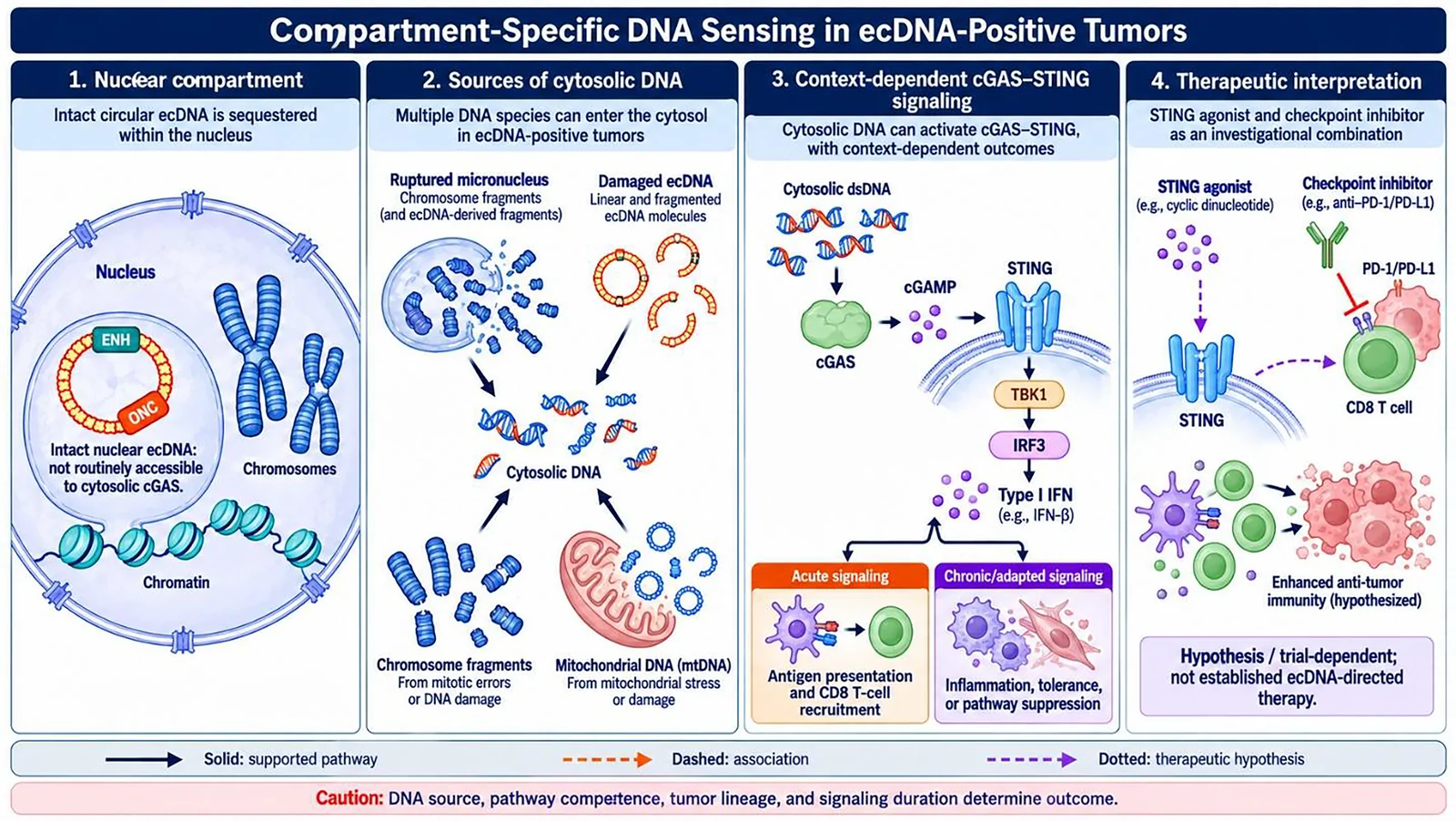
Compartment-specific DNA sensing in ecDNA-positive tumors. Intact ecDNA is predominantly retained within the nucleus and should not be considered a routine direct ligand for cytosolic cyclic GMP–AMP synthase (cGAS). Cytosolic DNA in ecDNA-positive tumors may instead originate from ruptured micronuclei, damaged ecDNA-derived fragments, conventional chromosome fragments, mitochondrial DNA, or other consequences of genomic instability. Cytosolic double-stranded DNA can activate the cGAS–cGAMP–STING–TBK1–IRF3 pathway and induce type I interferon signaling. Acute signaling may enhance antigen presentation and CD8-positive T-cell recruitment, whereas chronic or adapted signaling may promote inflammation, immune tolerance, or pathway suppression. STING agonists combined with immune-checkpoint inhibitors are shown as an investigational strategy rather than an established ecDNA-directed treatment. Solid arrows denote supported pathways, dashed arrows denote associations, and dotted arrows denote therapeutic hypotheses. Outcomes depend on DNA source, pathway competence, tumor lineage, cellular compartment, and signaling duration. Schematic prepared specifically for this review; not drawn to scale.

### 7.3 Oncogene-driven immunosuppression

The immune phenotype of an ecDNA-positive tumor may depend substantially on the biological activity of the genes carried by its ecDNA. For example, amplified *MYC* can suppress interferon responses, alter cytokine and chemokine expression, impair antigen presentation, and promote an immunosuppressive microenvironment. Amplification of *EGFR*, *MYCN*, *MDM2*, and other ecDNA-associated genes may similarly affect immune-cell recruitment, tumor-antigen presentation, inflammatory signaling, or immune-checkpoint expression. Immune escape may therefore result from cooperation between ecDNA architecture and ecDNA cargo. The circular structure increases gene dosage and facilitates strong transcription, while the amplified oncogene actively remodels tumor–immune interactions (55, 56). This distinction matters because immune suppression may not stem from ecDNA itself in every tumor; instead, ecDNA may amplify the immunoregulatory effects of the genes it carries.

### 7.4 Immune-checkpoint regulation

ecDNA may influence immune-checkpoint pathways through several direct and indirect mechanisms. Amplified receptor tyrosine kinases can activate downstream pathways that increase programmed death-ligand 1 expression, while ecDNA-associated *MYC* amplification may regulate immune-checkpoint genes and suppress antitumor immune responses. Enhancer rewiring or amplification of immune-regulatory loci could also alter PD-L1 and other checkpoint molecule expression. These mechanisms suggest that ecDNA status could affect responses to immune-checkpoint inhibitors (55–57). However, current evidence is insufficient to establish ecDNA as an independent predictive biomarker. Its apparent relationship with immunotherapy response may be confounded by tumor type, oncogene identity, tumor mutational burden, MHC status, interferon signaling, and immune microenvironment composition. Prospective clinical studies are needed to determine whether ecDNA detection adds predictive value beyond established biomarkers (25) (**Table 3**).

**Table 3:** Evidence hierarchy for proposed relationships between ecDNA and tumor immune escape.

Immune mechanism	Current finding	Attribution to ecDNA	Evidence level	Principal limitation
Reduced MHC class I and II programs	Some ecDNA-positive tumors show reduced transcription of genes involved in antigen processing and presentation	ecDNA-associated rather than proven ecDNA-specific	Level B	Mainly retrospective transcriptomic evidence; tumor lineage, purity, oncogene cargo, and interferon status may confound the association
Impaired cGAS–STING signaling	Reduced DNA-sensing or interferon activity has been observed in some ecDNA-positive settings.	May reflect selection caused by genomic instability rather than direct sensing of intact ecDNA	Level B–C	Intact ecDNA is predominantly nuclear; cytosolic DNA may originate from ruptured micronuclei, damaged ecDNA fragments, chromosomes, or mitochondria.
Oncogene-driven immune suppression	Amplified MYC, EGFR, MYCN, MDM2, and related drivers can alter interferon signaling, antigen presentation, and immune-cell recruitment.	Primarily an oncogene-cargo effect that ecDNA may intensify through increased dosage or regulatory rewiring	Level C for ecDNA specificity	Evidence for the oncogenes may be strong, but it does not establish that extrachromosomal topology independently causes the immune phenotype.
Reduced chemokine production and immune-cell exclusion	Some ecDNA-positive tumors exhibit immune-poor transcriptional or spatial profiles.	Context-dependent association	Level B–C	CXCL9, CXCL10, hypoxia, stromal signaling, and immune infiltration vary substantially by tumor lineage and microenvironment
ecDNA-driven cellular heterogeneity	Unequal segregation generates subclones with different ecDNA copy numbers and transcriptional states.	Direct ecDNA-specific evolutionary mechanism	Level A for heterogeneity; Level C for immune escape	Differences in antigen expression or immune recognition have not been demonstrated consistently
Reversible antigen loss	Selection could favor cells that reduce ecDNA carrying an immunogenic or therapeutically targeted gene.	Concept extrapolated from reversible loss of drug-targeted ecDNA	Level D for immune selection	Direct evidence that immune pressure causes reversible loss of antigen-bearing ecDNA is currently insufficient.
Immune-checkpoint regulation	ecDNA-borne oncogenes or regulatory elements may alter PD-L1 and related pathways	Primarily cargo-mediated or indirect	Level C–D	No universal checkpoint-amplification pattern and no validated ecDNA-specific predictive effect
Myeloid-cell or regulatory T-cell enrichment	Altered cytokine programs may promote tumor-associated macrophages, myeloid-derived suppressor cells, or regulatory T cells.	Indirect and tumor-context dependent	Level C–D	Requires spatial, functional, and tumor-specific validation
Chronic DNA-damage signaling	Persistent genomic instability may shift acute immune activation toward chronic inflammation or immune tolerance.	General chromosomal-instability mechanism relevant to some ecDNA-positive tumors	Level C	Acute and chronic cGAS–STING signaling may have opposite biological effects
Viral–human hybrid ecDNA	HPV–human hybrid ecDNA can sustain high *E6* and *E7* expression through rearranged regulatory architecture	Direct evidence for viral-oncogene regulation in selected HPV-associated models	Level B	Viral-antigen recognition and immune escape depend on additional host and tumor mechanisms and cannot be generalized to nonviral ecDNA

Evidence levels: Level A, direct ecDNA-specific evidence supported by structural confirmation and functional or longitudinal analysis; Level B, ecDNA-associated evidence from validated experimental models or clinical cohorts without definitive causal isolation; Level C, evidence derived mainly from oncogene amplification, genomic instability, or general cancer biology; Level D, conceptual or insufficiently tested ecDNA-specific hypothesis.

cGAS, cyclic GMP–AMP synthase; CXCL, C-X-C motif chemokine ligand; ecDNA, extrachromosomal DNA; HPV, human papillomavirus; MDM2, mouse double minute 2 homolog; MHC, major histocompatibility complex; MYC, MYC proto-oncogene; MYCN, MYCN proto-oncogene; PD-L1, programmed death-ligand 1; STING, stimulator of interferon genes.

### 7.5 Tumor microenvironment

Pan-cancer and tumor-specific studies have associated ecDNA positivity with reduced cytotoxic lymphocyte infiltration and other immune-suppressed transcriptional or spatial profiles (50, 54). These observations suggest that ecDNA status may correlate with tumor microenvironment composition, but they do not establish that ecDNA directly causes immune-cell exclusion. Several mechanisms could contribute to the observed association. Oncogenes carried on ecDNA may alter chemokine production, antigen presentation, interferon signaling, cellular metabolism, angiogenesis, or stromal interactions. Amplified *MYC*, *EGFR*, *MYCN*, and related drivers can produce immunoregulatory effects regardless of whether their amplification is extrachromosomal or chromosomal. Hypoxia, nutrient competition, vascular abnormalities, tumor purity, and recruitment of tumor-associated macrophages, myeloid-derived suppressor cells, or regulatory T cells may also influence immune infiltration. These mechanisms should therefore be described as cargo-mediated or microenvironment-dependent unless ecDNA topology is directly tested. The relationship varies across tumor types. Some ecDNA-positive tumors remain immune-infiltrated, particularly when they retain functional antigen presentation and innate DNA sensing or experience substantial immunogenic damage. Differences among published studies may reflect tumor lineage, amplified cargo, ecDNA burden, treatment history, sampling site, detection method, and the use of bulk rather than spatially resolved measurements. Accordingly, ecDNA should not be regarded as a universal marker of an immunologically cold tumor. Its immune significance should be evaluated together with oncogene identity, human leukocyte antigen status, interferon competence, stromal composition, and spatial immune organization. The evidence therefore supports an ecDNA-associated immune phenotype in selected settings, not a universal immune-suppressive program intrinsic to all ecDNA. **Figure 2** explicitly separates intact nuclear ecDNA from micronuclear and fragmented cytosolic DNA, and **Table 3** identifies where causality remains unproven.

## 8 Resistance to targeted therapy

The strongest evidence for ecDNA-mediated therapeutic adaptation comes from experimental models in which treatment changes the abundance of ecDNA carrying the drug target. In glioblastoma, exposure to an epidermal growth factor receptor tyrosine-kinase inhibitor selected cells with reduced extrachromosomal copies of mutant *EGFR*. After treatment withdrawal, *EGFR*-containing ecDNA re-emerged, restoring oncogenic signaling and the original drug-sensitive state (25, 56, 58). This reversible change provides direct evidence that ecDNA copy-number plasticity can support adaptation without requiring a new nucleotide-level resistance mutation. Related studies involving *BRAF* and MYC-family amplifications indicate that ecDNA copy number or structure can change under targeted-therapy pressure. However, these findings should not be generalized to every amplified oncogene or therapeutic agent. In some tumors, resistance may instead arise through persistent target amplification, intrachromosomal amplification, target mutation, bypass-pathway activation, lineage switching, or non-genetic cellular plasticity.

Several ecDNA-related processes may contribute to resistance in selected settings. These include reduced or lost ecDNA carrying the inhibited target, increased target copy number sufficient to overcome drug exposure, amplification of bypass-pathway genes, coexistence of functionally distinct ecDNA species, enhancer remodeling, chromosomal reintegration, and selection of pre-existing ecDNA-high or ecDNA-low subpopulations. Each mechanism requires longitudinal structural evidence before it can be classified as ecDNA-mediated. The therapeutic implication is not that every ecDNA-positive tumor requires the same combination treatment. Validated oncogenic drivers and tumor indications should guide treatment first. Experimental combinations may then target a documented bypass pathway or an ecDNA-associated dependency. Prospective studies should compare ecDNA-positive tumors with copy-number-matched tumors carrying intrachromosomal amplification and should assess ecDNA structure before treatment, during response, and at progression. As summarized in **Table 4** and **Figure 3**, targeted therapy currently provides the clearest direct evidence of ecDNA-enabled treatment adaptation. Among resistance phenotypes, selected targeted-therapy models provide the clearest demonstration that dynamic ecDNA abundance or structure can change with treatment and contribute to reversible adaptation.

**Table 4:** Evidence for ecDNA involvement in resistance to major anticancer treatments.

Treatment modality	ecDNA-associated observation or proposed mechanism	Representative examples	Evidence category	Interpretation and limitation
Targeted therapy	Reversible reduction, loss, or re-emergence of ecDNA carrying the inhibited target	*EGFR/EGFRvIII* in glioblastoma; *BRAF* amplification in selected models	Level A–B	Strongest ecDNA-specific resistance evidence, but demonstrated only for selected targets and models.
Targeted therapy	Increased target dosage or amplification of a bypass pathway may maintain signaling during treatment.	*EGFR*, *MET*, *FGFR2*, *ERBB2*, and MYC-family genes	Level B–C	Requires physical confirmation that the relevant amplification is extrachromosomal
Targeted therapy	Coexistence or coordinated inheritance of distinct ecDNA species may preserve complementary oncogenic pathways.	Multiple driver-bearing ecDNA species in selected ecDNA-rich tumors	Level B	Mechanistically plausible and experimentally supported in some models; clinical frequency and treatment implications remain uncertain.
Antimetabolite chemotherapy	Extrachromosomal *DHFR* amplification can emerge during methotrexate selection.	*DHFR* in methotrexate-resistant experimental systems	Level B	Established historical association; clinical relevance to contemporary treatment requires careful qualification.
Other cytotoxic chemotherapy	ecDNA carrying repair, survival, metabolic, or drug-response genes may be selected during treatment	*RAB3B*, *MYC*, *CCNE1*, and context-specific resistance genes	Level B–C	Cargo- and drug-dependent; amplification alone does not prove ecDNA-mediated resistance
Radiotherapy	Amplified repair or survival genes and ecDNA-associated heterogeneity may favor resistant clones.	*EGFR*, MYC-family genes, and DNA-damage-response pathways	Level C	Direct ecDNA-specific evidence is limited; ecDNA is not a validated marker of radioresistance.
Radiotherapy and innate immunity	Radiation-generated micronuclear or fragmented cytosolic DNA may activate cGAS–STING signaling	cGAS, STING, TBK1, IRF3, and type I interferon pathways	Level C	This is primarily a general radiation and genomic-instability mechanism; intact nuclear ecDNA should not be identified as the directly sensed ligand.
Immune-checkpoint inhibition	Reduced antigen-presentation programs and altered immune infiltration have been associated with ecDNA positivity.	MHC class I and II, interferon-response pathways, and oncogene-dependent immune programs	Level B–C	No prospective evidence validates ecDNA as an independent predictor of checkpoint-inhibitor resistance.
Monoclonal antibodies and cellular therapy	Unequal ecDNA inheritance could generate heterogeneous expression of an ecDNA-encoded surface antigen.	*EGFR*, *ERBB2*, or another tumor-associated surface target	Level D	Antigen loss caused specifically by ecDNA dynamics remains largely hypothetical.
BET and transcriptional inhibition	Altered enhancer architecture or transcriptional dependence may support adaptation.	BRD4, MYC-family genes, and HPV–human hybrid ecDNA	Level B–C	Direct preclinical evidence exists in selected contexts, particularly viral–human hybrid ecDNA; broad ecDNA selectivity is unproven.
DNA-damage-response inhibition	Alternative repair or checkpoint activity could modify treatment response in replication-stressed tumors.	ATR–CHK1, WEE1, DNA-PK, and homologous-recombination pathways	Level C	These pathways are not specific to ecDNA and require comparative validation in ecDNA-positive versus ecDNA-negative tumors.
Antiangiogenic or metabolic therapy	ecDNA-associated heterogeneity may facilitate adaptation to hypoxia or nutrient limitation	MYC-family, *EGFR*, and metabolic programs	Level D	Primarily conceptual; direct ecDNA-specific resistance evidence is insufficient
Epigenetic therapy	Persistent gene dosage or alternative regulatory elements may limit transcriptional repression.	MYC-family genes and other ecDNA-encoded transcriptional drivers	Level C–D	Mechanistic rationale exists, but prospective ecDNA-specific evidence is lacking.

Evidence levels: Level A, direct ecDNA-specific evidence supported by structural confirmation and functional or longitudinal analysis; Level B, ecDNA-associated evidence from validated experimental models or clinical cohorts without complete causal isolation; Level C, evidence derived mainly from amplified oncogene biology, genomic instability, or broader cancer mechanisms; Level D, conceptual or insufficiently tested ecDNA-specific hypothesis.

ATR, ataxia telangiectasia and Rad3-related protein; BET, bromodomain and extraterminal domain; BRD4, bromodomain-containing protein 4; CAR-T, chimeric antigen receptor T-cell; cGAS, cyclic GMP–AMP synthase; CHK1, checkpoint kinase 1; DHFR, dihydrofolate reductase; ecDNA, extrachromosomal DNA; EGFR, epidermal growth factor receptor; ERBB2, erb-b2 receptor tyrosine kinase 2; FGFR2, fibroblast growth factor receptor 2; HPV, human papillomavirus; MHC, major histocompatibility complex; STING, stimulator of interferon genes; TBK1, TANK-binding kinase 1; WEE1, WEE1 G2 checkpoint kinase.

**Figure 3:**
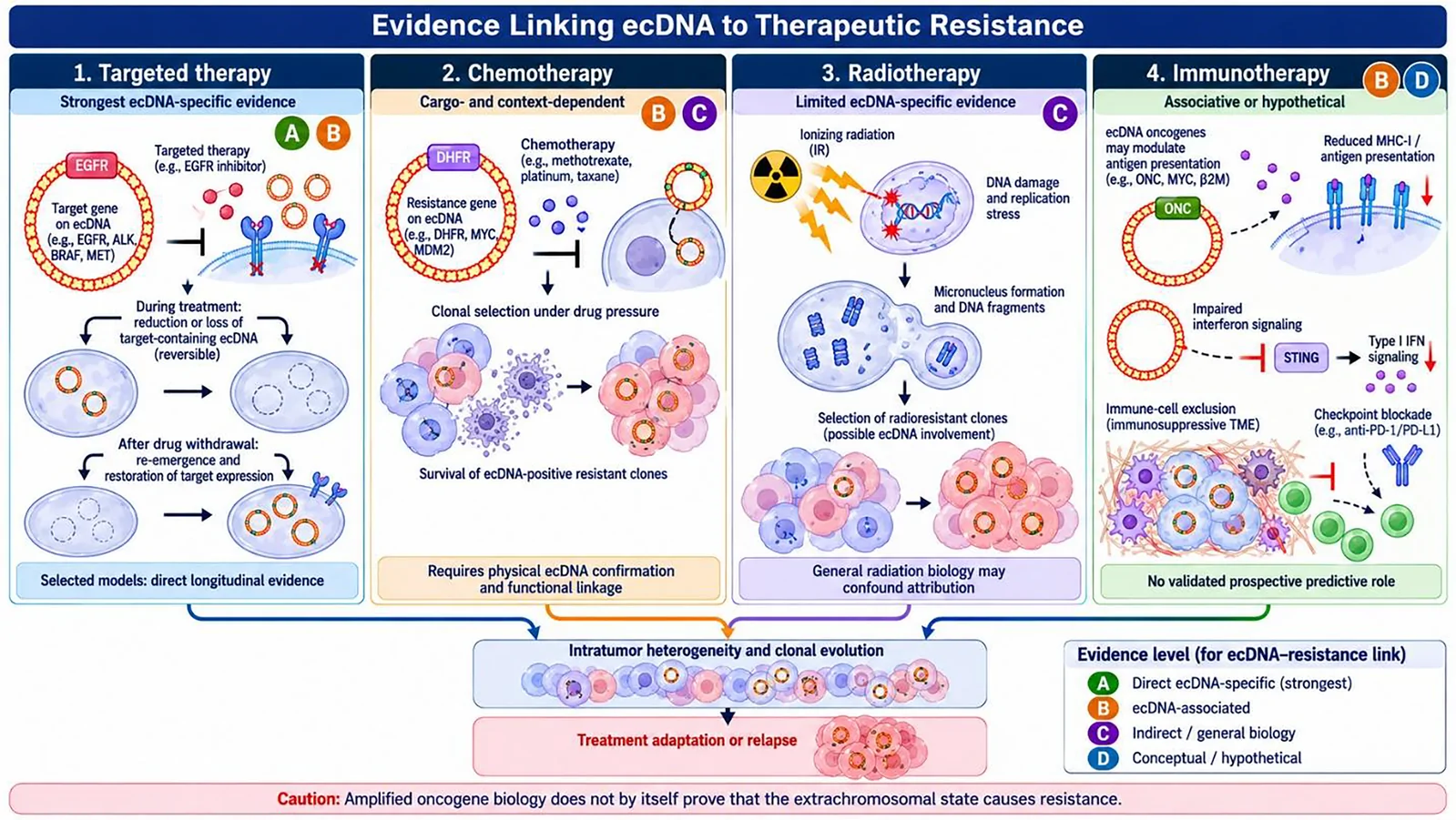
Evidence linking ecDNA to resistance across major anticancer treatment modalities. Selected targeted-therapy models provide the strongest ecDNA-specific evidence, including reversible reduction or loss of target-containing ecDNA during treatment and re-emergence after drug withdrawal. Chemotherapy resistance may involve amplification of resistance-associated genes on ecDNA and treatment-mediated clonal selection, but attribution requires physical confirmation of ecDNA and functional linkage to drug response. Proposed contributions to radiotherapy resistance remain difficult to distinguish from general DNA-damage responses, replication stress, micronuclear formation, and chromosomal instability. Associations with immunotherapy resistance may involve oncogene-cargo-mediated suppression of antigen presentation, altered interferon signaling, or immune-cell exclusion; however, a prospective predictive role remains unvalidated. These processes may converge on intratumor heterogeneity, clonal evolution, treatment adaptation, and relapse. Evidence levels are defined as A, direct ecDNA-specific evidence; B, ecDNA-associated evidence; C, indirect evidence derived from amplified-oncogene or general cancer biology; and D, conceptual or hypothetical evidence. Amplified-oncogene biology alone does not demonstrate that the extrachromosomal state causes resistance. Schematic prepared specifically for this review; not drawn to scale.

## 9 Chemotherapy resistance

The historical association between double minutes and dihydrofolate reductase amplification during methotrexate selection provides an early example of extrachromosomal amplification accompanying chemotherapy resistance. More recent studies suggest that ecDNA may carry genes involved in drug transport, nucleotide metabolism, detoxification, DNA repair, cell-cycle regulation, autophagy, or apoptosis. When such cargo increases survival under a particular drug, chemotherapy can select cells carrying the advantageous ecDNA structure. Unequal ecDNA inheritance may increase the range of copy-number states present before treatment. Chemotherapy can eliminate sensitive populations while allowing cells with favorable ecDNA copy number or cargo to expand. In this situation, apparent acquired resistance may represent selection of pre-existing heterogeneity rather than the creation of an entirely new resistance alteration. Nevertheless, demonstrating ecDNA-mediated resistance requires evidence that the relevant resistance gene is physically located on ecDNA and that changes in its ecDNA copy number or expression affect drug response.

ecDNA does not intrinsically confer chemotherapy resistance. High-copy and strongly transcribed ecDNA can increase replication burden, transcription–replication conflicts, and DNA damage. These features may sensitize some ecDNA-positive cells to DNA-damaging agents or replication-stress-directed treatments. The direction of the response therefore depends on the drug, ecDNA cargo, copy number, repair capacity, cellular background, and the fitness costs of maintaining the amplified molecule. Many reported chemotherapy-resistance mechanisms are based on amplification generally rather than confirmed ecDNA. Studies should therefore distinguish extrachromosomal from intrachromosomal amplification and include copy-number-matched controls. Current evidence supports a cargo- and context-dependent role for ecDNA in selected chemotherapy-resistance models, not a universal chemoresistant phenotype. Chemotherapy resistance should therefore be attributed to ecDNA only when the relevant resistance cargo is physically localized to ecDNA and longitudinal or functional data connect its change to drug response.

## 10 Radiotherapy response and resistance

Direct ecDNA-specific evidence for radiotherapy resistance remains limited. Radiotherapy produces DNA double-strand breaks, oxidative damage, replication stress, micronuclei, and mitotic abnormalities. These effects can select heterogeneous tumor-cell populations, but they are not specific to ecDNA-positive cancers. Amplification of DNA-repair, cell-cycle, survival, or anti-apoptotic genes on ecDNA could provide a cargo-mediated mechanism of radioresistance. Unequal segregation may also increase the probability that clones with favorable copy-number states exist before treatment. However, these hypotheses require confirmation that the relevant genes are carried on ecDNA and that changes in ecDNA abundance or structure alter radiation response. Amplification of a repair gene without evidence of extrachromosomal localization should not be classified as an ecDNA-specific mechanism. Conversely, the replication stress and genomic instability associated with some ecDNA-positive tumors may increase sensitivity to radiation or DNA-damage-response inhibition. The net effect is therefore likely to vary by tumor lineage, ecDNA cargo, baseline repair capacity, radiation schedule, and treatment history. ecDNA should not presently be considered a validated biomarker of radiosensitivity or radioresistance. Radiation-induced micronuclei and cytosolic DNA can activate cyclic GMP–AMP synthase–stimulator of interferon genes signaling. The sensed DNA may originate from damaged chromosomes, ruptured micronuclei, mitochondria, or ecDNA-derived fragments; it should not automatically be described as intact ecDNA. Acute pathway activation may promote type I interferon production and antitumor immunity, whereas chronic signaling may support inflammation, immune tolerance, or metastatic behavior. Proposed combinations involving radiotherapy, stimulator of interferon genes agonists, checkpoint inhibitors, or ATR, CHK1, and WEE1 inhibitors therefore remain investigational. Their evaluation requires ecDNA-stratified models and careful consideration of dose, fractionation, sequence, pathway competence, and toxicity. For radiotherapy, ecDNA is currently better viewed as a candidate context marker linked to replication stress and genomic instability than as a validated, independent predictor of radiosensitivity or radioresistance.

## 11 Potential association with immunotherapy resistance

Direct prospective clinical evidence linking ecDNA to failure of immune-checkpoint inhibition is currently insufficient. Some ecDNA-positive tumors exhibit reduced antigen-presentation transcripts, altered interferon signaling, or diminished immune-cell infiltration, providing a plausible rationale for reduced immunotherapy activity (50, 54). However, these features do not establish ecDNA as an independent predictive biomarker. Several potential mechanisms have been proposed. An oncogene amplified on ecDNA may suppress antigen presentation, alter chemokine expression, increase checkpoint-ligand expression, or exclude cytotoxic lymphocytes. Unequal segregation may generate subclones with different levels of an immunoregulatory or antigen-associated gene. Persistent genomic instability may select for impaired innate DNA sensing. These mechanisms differ in evidence strength. Oncogene-mediated immune regulation is supported in relevant cancer models, whereas immune selection causing reversible loss of antigen-bearing ecDNA remains largely hypothetical.

Retrospective or machine-learning associations between ecDNA-related signatures and immune phenotypes require cautious interpretation. Computational signatures may identify focal amplification or chromosomal instability without physically confirming ecDNA. Observed relationships may also be confounded by tumor lineage, oncogene identity, tumor mutational burden, human leukocyte antigen status, interferon competence, prior treatment, and immune-cell composition. Prospective immunotherapy studies should define ecDNA using breakpoint-resolved reconstruction and, where feasible, orthogonal structural confirmation. Analyses should include ecDNA-negative tumors with comparable driver amplification, adjust for established immunotherapy biomarkers, and test whether ecDNA status improves prediction beyond these variables. Serial tissue or tumor-informed plasma analysis may help determine whether immunotherapy alters ecDNA abundance or clonal structure. Until such evidence is available, ecDNA should be described as a candidate biomarker for immunotherapy research rather than a clinically actionable predictor of immune-checkpoint inhibitor resistance. **Figure 3** therefore presents the immunotherapy relationship as associative or hypothetical, in contrast to the more direct evidence available for selected targeted-therapy models. Accordingly, immunotherapy resistance occupies the associative-to-hypothetical end of the evidence hierarchy and should not be used for clinical treatment selection outside prospective studies.

## 12 Therapeutic vulnerabilities of ecDNA-positive cancers

The biological properties that support ecDNA-mediated amplification and tumor evolution may also create therapeutic liabilities. Proposed strategies fall into three categories: targeting an oncogenic driver carried on ecDNA; exploiting a cellular dependency demonstrated in an ecDNA-positive model; and interfering with a theoretical requirement for ecDNA maintenance, inheritance, or immune adaptation. These categories are not equivalent. Targeting an amplified oncogene may already be clinically appropriate when a validated drug, biomarker, and tumor indication exist. In such cases, however, clinical actionability derives from the oncogenic alteration and disease context rather than from its location on ecDNA. By contrast, therapies specifically selected because a tumor is ecDNA-positive remain investigational. Among these approaches, replication-stress enhancement and CHK1 inhibition have the strongest direct preclinical support. ATR, WEE1, transcriptional inhibitors, STING agonists, ecDNA-hub disruption, inheritance blockade, and CRISPR-based approaches have more limited or context-dependent evidence (45, 51, 59, 60).

### 12.1 Replication stress

Replicating numerous ecDNA copies can impose a substantial DNA-synthesis burden. ecDNA may also support intense transcription, increasing the probability of collisions between replication forks and transcriptional machinery. These transcription–replication conflicts can cause fork stalling, fork collapse, incomplete replication, DNA breakage, and activation of cell-cycle checkpoints. Direct experimental evidence indicates that enhancing transcription–replication conflict can preferentially damage ecDNA-positive cancer models (45). Nevertheless, replication stress is common in many ecDNA-negative cancers, including tumors with MYC-family activation, defective DNA repair, or rapid proliferation. Replication stress should therefore not be considered an ecDNA-specific vulnerability without an appropriate comparator. To establish predictive specificity, studies should compare ecDNA-positive tumors with ecDNA-negative tumors matched for oncogenic driver, amplification level, tumor lineage, and baseline replication-stress markers. Such comparisons are necessary to determine whether ecDNA independently predicts sensitivity or merely identifies tumors that already exhibit a broader replication-stress phenotype.

### 12.2 CHK1 inhibition

Checkpoint kinase 1 coordinates the cellular response to replication stress by stabilizing stalled replication forks, restraining inappropriate origin firing, promoting DNA repair, and delaying cell-cycle progression. These functions may be particularly important in ecDNA-positive cells with high replication and transcriptional burdens. Preclinical studies have shown that increasing transcription–replication conflict while inhibiting CHK1 can preferentially damage selected ecDNA-positive models (45). Combining CHK1 inhibition with an agent directed against the ecDNA-encoded oncogene produced greater and more sustained tumor control than either intervention alone in these experimental systems. This represents direct ecDNA-specific preclinical evidence, although it does not establish clinical efficacy or universal sensitivity across ecDNA-positive tumors. BBI-355 is an orally administered CHK1 inhibitor undergoing phase I/II clinical evaluation in patients with tumors containing oncogene amplification on ecDNA (ClinicalTrials.gov identifier NCT05827614). Clinical-trial enrollment and preliminary conference reports should not be taken as evidence of established efficacy. Peer-reviewed comparative results are required to determine safety, therapeutic activity, and whether ecDNA predicts response independently of oncogene identity, copy number, and replication-stress status. CHK1 inhibition should therefore be described as the most clinically advanced investigational ecDNA-directed strategy, not as an established treatment.

### 12.3 ATR and WEE1 inhibition

Ataxia telangiectasia and Rad3-related protein and WEE1 kinase regulate replication stress and cell-cycle integrity. Ataxia telangiectasia and Rad3-related protein respond to replication-associated single-stranded DNA and stabilize stalled forks, whereas WEE1 limits premature mitotic entry by restraining cyclin-dependent kinase activity. Inhibiting these proteins can force cells with incompletely replicated or damaged DNA through the cell cycle, causing replication catastrophe or mitotic death. The elevated replication burden of some ecDNA-positive tumors provides a rationale for ATR or WEE1 inhibition, alone or in combination with oncogene-directed therapy, radiotherapy, or DNA-damaging chemotherapy. However, these dependencies are not unique to ecDNA. Researchers are studying ATR and WEE1 inhibitors across multiple cancers selected by replication stress, DNA-repair defects, or oncogene activation. Current support for ATR and WEE1 inhibition as specifically ecDNA-directed therapy is therefore indirect and context-dependent. Comparative studies must determine whether ecDNA status adds predictive value beyond established replication-stress and DNA-repair biomarkers. Until such evidence is available, these agents should not be presented as validated treatments for ecDNA-positive cancers.

### 12.4 Nucleotide metabolism

High-copy DNA replication requires a sustained supply of deoxyribonucleotides. This creates a plausible dependence on ribonucleotide reductase, nucleotide-synthesis enzymes, salvage pathways, and metabolic processes that maintain nucleotide balance. Restricting nucleotide availability could increase fork stalling and DNA damage in cells replicating numerous ecDNA copies. This hypothesis has not yet been shown to be broadly selective for ecDNA. Rapidly proliferating ecDNA-negative tumors and normal tissues, particularly bone marrow and intestinal epithelium, also depend on nucleotide synthesis. Proposed combinations of nucleotide depletion with CHK1, ATR, or WEE1 inhibition may therefore produce substantial toxicity. Nucleotide-metabolism targeting should be classified as an indirect mechanistic rationale until ecDNA-controlled studies demonstrate a therapeutic window. Future investigations should compare ecDNA-positive and matched ecDNA-negative models and assess whether sensitivity depends on ecDNA burden, transcriptional activity, replication timing, or the identity of the amplified driver.

### 12.5 Transcriptional dependencies

Many ecDNA molecules contain active enhancers and promoters that support high expression of amplified oncogenes. This regulatory activity supports inhibition of bromodomain and extraterminal proteins, including BRD4, CDK7, CDK9, histone acetyltransferases, lineage-specific transcription factors, and other proteins involved in enhancer-dependent transcription. Direct preclinical evidence is strongest in selected HPV–human hybrid ecDNA models, in which disruption of ecDNA-associated enhancers or inhibition of enhancer-related transcription reduced viral-oncogene expression and tumor growth (51). This finding demonstrates a regulatory dependency in a defined viral–human ecDNA context. It does not establish that all ecDNA-positive tumors are selectively sensitive to BET, CDK7, or CDK9 inhibition. Transcriptional regulators control essential genes in malignant and normal cells, and ecDNA transcription is not uniform across tumor types. Some studies support cooperative transcription within ecDNA hubs, whereas others suggest that copy number alone explains high oncogene expression. Sensitivity therefore likely depends on ecDNA architecture, enhancer composition, oncogene cargo, and tumor lineage. These strategies remain preclinical as ecDNA-directed treatments.

### 12.6 Disrupting ecDNA hubs

ecDNA hubs can support cooperative enhancer–promoter interactions in selected experimental systems. Disrupting hub formation, transcriptional condensates, intermolecular enhancer contacts, or RNA polymerase recruitment could therefore reduce oncogene expression without physically eliminating ecDNA. This approach remains conceptual because no molecular component is uniquely required for ecDNA hubs across cancers. Proteins involved in condensate formation, enhancer activity, and nuclear organization also regulate chromosomal transcription. Inhibiting them may therefore affect both ecDNA-positive and ecDNA-negative cells and cause substantial toxicity. Evidence is further complicated by studies showing that spatial clustering is not required for high oncogene expression in some ecDNA-positive models. A candidate hub-disrupting therapy should demonstrate loss of ecDNA-specific contacts, reduced expression of ecDNA-encoded genes, and greater activity against ecDNA-positive cells than against copy-number-matched chromosomal-amplification controls. Until these criteria are met, hub disruption should be described as a hypothesis rather than an established therapeutic strategy.

### 12.7 Blocking ecDNA replication or inheritance

For ecDNA to persist across cell divisions, it must replicate, remain within the nucleus, and be retained during mitosis. Recent studies have identified sequence elements, chromosomal associations, and tethering processes that contribute to ecDNA nuclear retention and inheritance (59, 60). These findings establish biological mechanisms that could eventually provide therapeutic targets. Therapeutic interference with ecDNA replication origins, replication-licensing factors, nuclear-retention elements, or mitotic tethering remains early preclinical or conceptual. Most candidate factors also perform essential functions on conventional chromosomes. Selective inhibition will therefore require targets that affect ecDNA more strongly than chromosomal DNA. Forcing a reduction in ecDNA may also create new evolutionary pressures. Tumor cells could reintegrate the amplified material into chromosomes, develop alternative amplification structures, or select ecDNA-independent subclones. Future experiments should assess both immediate ecDNA depletion and subsequent structural evolution. Longitudinal genomic monitoring will be essential before this strategy is clinically applicable.

### 12.8 CRISPR-based targeting

CRISPR-based research has already contributed to ecDNA detection and functional analysis. CRISPR-CATCH has been used to isolate and structurally characterize ecDNA molecules, while CRISPR interference directed against enhancers on HPV–human hybrid ecDNA has reduced oncogene expression and tumor growth in experimental models (18, 51). These applications demonstrate the value of CRISPR technology for ecDNA profiling and transcriptional perturbation. Therapeutic cleavage of patient-specific ecDNA breakpoint junctions has not been established clinically. Cas9-mediated junction cleavage, base editing, prime editing, targeted recruitment of DNA-degradation machinery, and systemic elimination of high-copy ecDNA remain conceptual or early preclinical approaches. A tumor-specific breakpoint provides a potential target but does not prove it can be edited safely or efficiently in patients. Major barriers include ecDNA copy-number heterogeneity, structural diversity among tumor cells, delivery to solid tumors, incomplete editing, off-target effects, immune responses to editing components, and the possibility that double-strand cleavage could generate new rearrangements or promote chromosomal reintegration. Non-cleaving CRISPR interference may reduce the risk of DNA-break-associated rearrangement, but delivery and durability remain unresolved. CRISPR-based ecDNA targeting should therefore be presented as an experimental research strategy rather than an established treatment.

### 12.9 Restoring innate immune sensing

Some ecDNA-positive tumors exhibit reduced DNA-sensing or interferon-related transcriptional programs, suggesting that restoration of innate immunity could be therapeutically relevant. Proposed approaches include STING agonists, epigenetic restoration of cGAS or STING expression, radiotherapy-mediated generation of cytosolic DNA, and combinations with immune-checkpoint inhibition. The rationale requires careful qualification. Intact ecDNA is predominantly nuclear and should not be assumed to activate cytosolic cGAS directly. DNA sensing in an ecDNA-positive tumor may instead involve ruptured micronuclei, damaged ecDNA-derived fragments, chromosomal fragments, mitochondrial DNA, or other cytosolic DNA species. It must also be established that the cGAS–STING pathway remains present and functionally responsive.

STING activation can have opposing effects. Acute activation may promote type I interferon production, dendritic-cell function, antigen presentation, and cytotoxic T-cell recruitment. Persistent signaling may instead support chronic inflammation, immune tolerance, metastasis, or tissue toxicity. The biological result depends on tumor lineage, pathway integrity, cellular compartment, dose, schedule, and route of administration. STING agonism is therefore an investigational cancer strategy with limited ecDNA-specific validation. Future studies should compare ecDNA-positive and matched ecDNA-negative models, define the cytosolic DNA source, and test whether ecDNA status independently predicts therapeutic response. Proposed combinations with radiotherapy or checkpoint blockade should not be presented as established ecDNA-directed treatments. **Figure 4** summarizes these strategies according to evidence strength. We distinguish direct ecDNA-specific preclinical evidence from indirect or context-dependent rationale and from conceptual or early preclinical approaches. Currently, no ecDNA-directed strategy is established for routine clinical use. The therapeutic story is therefore deliberately narrow: driver-directed treatment may be actionable because of the driver, CHK1-based exploitation of transcription–replication conflict has the strongest ecDNA-specific preclinical support, and all other ecDNA-directed concepts require additional selectivity and clinical validation.

**Figure 4:**
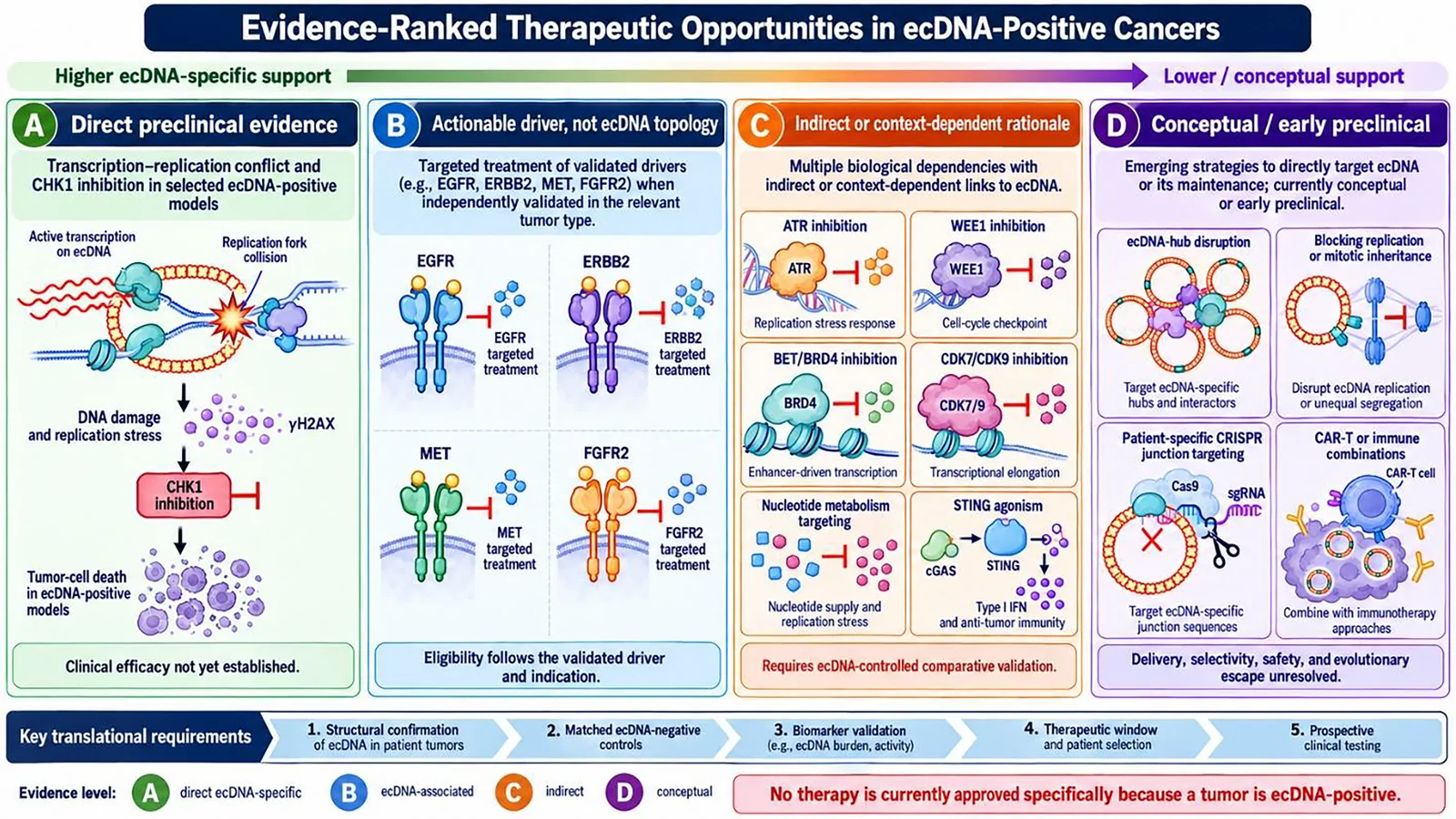
Evidence-ranked therapeutic opportunities in ecDNA-positive cancers. Strategies are arranged from higher to lower ecDNA-specific support. **(A)** Direct preclinical evidence: enhancing transcription -replication conflicts combined with checkpoint kinase 1 (CHK1) inhibition has shown activity in selected ecDNA-positive models; clinical efficacy has not been established. **(B)** Clinically actionable drivers: targeted treatment of EGFR, ERBB2, MET, or FGFR2 may be appropriate when independently validated in the relevant tumor type. Eligibility derives from the driver and indication rather than ecDNA topology. **(C)** Indirect or context-dependent rationale: ATR, WEE1, BET/BRD4, CDK7/CDK9, nucleotide-metabolism, and STING-directed strategies require ecDNA-controlled comparative validation. **(D)** Conceptual or early preclinical approaches: ecDNA-hub disruption, interference with ecDNA replication or mitotic inheritance, patient-specific CRISPR junction targeting, and CAR-T-cell or other immune combinations face unresolved delivery, selectivity, safety, and evolutionary-escape challenges. The lower pathway summarizes translational requirements: structural confirmation of ecDNA, matched ecDNA-negative controls, biomarker validation, demonstration of a therapeutic window, and prospective clinical testing. No therapy is currently approved specifically because a tumor is ecDNA-positive. Schematic prepared specifically for this review; not drawn to scale.

## 13 Detection and biomarker development

Clinical translation of ecDNA biology depends on accurate, reproducible identification in tumor tissue and body fluids. No single detection method currently captures all relevant characteristics, including circular topology, breakpoint structure, copy number, transcriptional activity, and cell-to-cell heterogeneity (61, 62). Copy-number amplification alone is insufficient to establish ecDNA because similar patterns can arise through intrachromosomal amplification or breakage–fusion–bridge cycles. An integrated approach combining cytogenetic imaging, sequencing-based reconstruction, and functional analysis therefore provides the strongest evidence for ecDNA.

### 13.1 Cytogenetic imaging

Metaphase fluorescence *in situ* hybridization can directly visualize extrachromosomal copies of selected genomic loci and distinguish them from amplification within conventional chromosomes. This method remains highly informative for validating ecDNA and determining its approximate cellular abundance. However, metaphase FISH requires actively dividing cells, technically suitable specimens, and prior knowledge of the amplified target. These requirements limit its application to fixed archival tissue and routine diagnostic workflows. Interphase FISH is more compatible with formalin-fixed tissue, cytological specimens, and routine pathological assessment. It can identify heterogeneous amplification patterns at the single-cell level but may not reliably distinguish ecDNA from tightly clustered intrachromosomal amplification (63–65). Other imaging approaches, including spectral karyotyping, DNA painting, super-resolution microscopy, and combined DNA–RNA FISH, may provide additional information about ecDNA localization, structure, copy number, and transcriptional activity. Nevertheless, imaging results should ideally be integrated with genomic evidence of circularization.

### 13.2 Whole-genome sequencing

Whole-genome sequencing can identify focal copy-number amplification, discordant read pairs, split reads, and breakpoint patterns compatible with circular amplicons. Computational tools such as AmpliconArchitect reconstruct amplified genomic segments and infer their structural relationships. At the same time, AmpliconClassifier categorizes reconstructed amplicons into ecDNA-like circular structures, breakage–fusion–bridge-associated amplification, linear amplification, or other complex configurations (65). These methods have enabled large pan-cancer studies of ecDNA prevalence and genomic composition. Sequencing-based inference remains sensitive to tumor purity, sequencing depth, subclonal abundance, repetitive DNA, and the complexity of the amplified region. Highly heterogeneous ecDNA populations may generate multiple competing structural reconstructions, and short-read sequencing may not resolve every breakpoint or prove that predicted segments coexist on the same physical circle (61, 62). Computationally inferred ecDNA should therefore be described cautiously unless imaging, long-read sequencing, optical mapping, or another orthogonal validation method supports it.

### 13.3 Long-read sequencing and optical genome mapping

Long-read sequencing can span complex breakpoint junctions, repetitive regions, and rearranged segments that short reads struggle to resolve. It can improve ecDNA reconstruction, phase variants across amplified regions, and determine whether regulatory elements and oncogenes are present on the same molecule. However, very large or structurally heterogeneous ecDNA molecules may remain difficult to reconstruct completely, particularly when coverage is limited (61, 62, 66). Optical genome mapping analyses high-molecular-weight DNA and can identify large structural rearrangements, segment order, and complex amplification patterns. Its resolution for small nucleotide-level junctions is lower than sequencing, but it provides valuable long-range structural information (67–69). Combining short-read sequencing, long-read sequencing, optical mapping, and cytogenetic imaging offers substantially stronger evidence than any individual method because it connects breakpoint-level resolution with physical confirmation of extrachromosomal localization.

### 13.4 Single-cell and spatial analysis

Bulk sequencing reports the average genomic state of a tumor and may therefore conceal substantial variation in ecDNA copy number and structure. Single-cell DNA sequencing and imaging can reveal the distribution of ecDNA across individual tumor cells, identify ecDNA-high and ecDNA-low subpopulations, and track their selection during treatment. Single-cell RNA sequencing can link ecDNA abundance to oncogene expression and cellular phenotype, whereas single-cell ATAC sequencing can characterize chromatin accessibility at ecDNA-associated regulatory elements (63, 65). Spatial profiling may determine whether ecDNA-rich populations preferentially occupy hypoxic, invasive, immune-excluded, or treatment-resistant tumor regions. Integrating spatial transcriptomics with DNA FISH, protein imaging, and single-cell genomic analysis could connect ecDNA architecture to oncogenic signaling, immune infiltration, and microenvironmental selection. Important technical challenges include sparse genomic coverage, amplification bias, difficulty assigning sequencing reads to circular structures, and loss of spatial information during tissue dissociation (61, 62).

### 13.5 Liquid biopsy

Tumor-specific ecDNA breakpoint junctions represent potentially valuable biomarkers because they may be absent from normal tissues and unique to an individual tumor. When these junction sequences are released into circulation, targeted assays could detect them within circulating tumor DNA. Potential clinical applications include: non-invasive identification of ecDNA-positive disease; longitudinal monitoring of ecDNA burden; detection of emerging resistant ecDNA structures; measurement of treatment-associated ecDNA reduction; assessment of minimal residual disease; and early detection of molecular relapse. Breakpoint-specific digital PCR, targeted deep sequencing, error-corrected sequencing, and personalized circulating tumor DNA assays could enable sensitive monitoring after the ecDNA structure is defined in the primary tumor. Serial sampling may be particularly valuable because ecDNA architecture and copy number can change rapidly under treatment pressure (62, 66). A major limitation is that most circulating tumor DNA consists of short, linear fragments. Detecting an ecDNA-derived breakpoint therefore does not show that an intact circular molecule is circulating in the blood. Assays must distinguish intact extrachromosomal circles from fragmented DNA released from tumor ecDNA and similar junctions produced by chromosomal rearrangements. Additional challenges include low tumor DNA fractions, variable DNA shedding, clonal hematopoiesis, and the lack of standardized analytical thresholds. For these reasons, liquid-biopsy ecDNA detection remains promising but requires prospective clinical validation and comparison with tissue-based imaging and genomic reconstruction. A clinically useful diagnostic hierarchy is thus: copy-number screening identifies candidates; breakpoint-resolved sequencing establishes probable ecDNA; and imaging, long-read sequencing, optical mapping, or another orthogonal method provides stronger physical confirmation. Liquid-biopsy junctions may monitor a tumor-defined structure but do not prove that intact ecDNA circulates.

## 14 A Precision-oncology framework

An ecDNA-directed precision-oncology strategy should extend beyond reporting gene amplification. Conventional copy-number analysis may identify the amplified gene but cannot reliably determine whether the amplification is extrachromosomal, intrachromosomal, or generated through breakage–fusion–bridge cycles (70–72). This distinction is clinically relevant because ecDNA is associated with unequal inheritance, rapid copy-number adaptation, enhancer rewiring, transcriptional hyperactivity, and dynamic responses to treatment. Comprehensive characterization should therefore integrate genomic structure, regulatory activity, cellular heterogeneity, immune phenotype, and longitudinal evolution. Clinical profiling of an ecDNA-positive tumor should determine: whether the amplification is extrachromosomal or chromosomal; which oncogenes, enhancers, non-coding RNAs, and immune-regulatory elements are carried by ecDNA (66); whether multiple structurally or functionally distinct ecDNA species coexist; how ecDNA copy number is distributed among individual tumor cells; whether the tumor displays replication-stress or transcriptional dependencies; the status of antigen presentation, cGAS–STING, and interferon signaling; whether ecDNA structure or abundance changes during treatment; and whether tumor-specific ecDNA breakpoint junctions can be monitored in plasma. An integrated workflow could begin with whole-genome sequencing and computational amplicon reconstruction, followed by imaging or long-read validation of ecDNA structure (73–76). Single-cell and spatial analyses could then define ecDNA heterogeneity and its association with resistant or immune-excluded tumor regions (77). Functional biomarkers of replication stress, enhancer dependence, DNA sensing, and antigen presentation may guide treatment selection. Finally, serial tissue or plasma analysis could detect ecDNA loss, structural remodeling, chromosomal reintegration, or the emergence of resistant ecDNA species **Table 5**.

**Table 5:** Evidence-ranked therapeutic strategies and biomarkers for ecDNA-positive cancers.

Strategy	What has been demonstrated	Evidence level and stage	Biomarker or selection approach	Clinical interpretation
Target an ecDNA-borne oncogenic driver.	Approved or investigational drugs may inhibit amplified drivers such as EGFR, ERBB2, MET, or FGFR2 in appropriate tumor types.	Clinical evidence may exist for the driver, but not necessarily for ecDNA topology.	Validated driver amplification or expression using an indication-specific clinical assay	Currently actionable only when the driver and tumor indication are independently validated; ecDNA is not yet the treatment-selection biomarker.
CHK1 inhibition with enhanced transcription–replication conflict	Preferential activity and improved combination responses have been demonstrated in selected ecDNA-positive experimental models.	Level A preclinical evidence; BBI-355 is under Phase I/II investigation	Structurally confirmed ecDNA, oncogene cargo, copy number, and replication-stress markers	Most advanced specifically ecDNA-directed approach, but clinical efficacy and predictive specificity remain investigational
ATR or WEE1 inhibition	Replication-stress and cell-cycle mechanisms provide a rationale for sensitivity.	Level C; preclinical ecDNA rationale and broader oncology trials	Replication-stress signature, phosphorylated RPA, DNA-repair defects, and ecDNA status	These dependencies are not unique to ecDNA; comparative validation is required
Nucleotide-metabolism inhibition	High-copy DNA replication may increase nucleotide requirements	Level C–D; mechanistic and early preclinical rationale	ecDNA burden and nucleotide-metabolism activity	Selectivity for ecDNA is unproven, and toxicity to normal proliferating tissues may limit treatment.
BET, BRD4, CDK7, or CDK9 inhibition	Enhancer or transcriptional inhibition can reduce oncogene expression in selected ecDNA models, including HPV–human hybrid ecDNA	Level B–C; preclinical	ecDNA enhancer architecture, BRD4 or H3K27ac occupancy, and ecDNA-derived transcription	Evidence is context-dependent; broad ecDNA selectivity and a therapeutic window have not been established
CRISPR interference of ecDNA enhancers	Repression of hybrid ecDNA enhancers reduced viral-oncogene expression and tumor growth in experimental HPV-associated models.	Level B; preclinical	Structurally confirmed hybrid ecDNA and functional enhancer–promoter mapping	Demonstrated in a defined viral–human ecDNA setting; delivery, durability, and applicability to nonviral ecDNA remain uncertain
CRISPR cleavage or editing of ecDNA junctions	Tumor-specific junctions can be identified and used for ecDNA profiling, but therapeutic editing has not been established clinically.	Level D; conceptual or early preclinical	Patient-specific breakpoint confirmed by whole-genome or long-read sequencing	High copy number, heterogeneity, off-target editing, delivery, and rearrangement risk remain major barriers.
ecDNA-hub disruption	Cooperative hub-associated transcription has been demonstrated in selected models.	Level D therapeutic evidence; discovery stage	Imaging of ecDNA clustering and functional chromatin-interaction assays	No validated hub-specific drug target exists, and some models do not require hubs for high oncogene expression
Replication, mitotic-tethering, or nuclear-retention blockade	Sequence elements and chromosomal associations involved in ecDNA retention and inheritance have been identified	Level B for biological mechanism; Level D for therapy	ecDNA-retention elements, tethering factors, and live-cell imaging	Therapeutic selectivity is unproven; intervention may select chromosomal reintegration or alternative amplification
STING agonism or restoration of innate sensing	STING activation has general anticancer rationale, but ecDNA-controlled therapeutic evidence is limited	Level C–D; investigational in broader cancer populations	Functional cGAS–STING pathway, interferon-low phenotype, and defined cytosolic DNA source	Intact nuclear ecDNA should not be assumed to activate cGAS directly; chronic signaling may be protumorigenic
Restoration of antigen presentation	Interferon or epigenetic treatment may increase MHC expression in pathway-competent tumors.	Level C–D; general preclinical or early clinical rationale	Low MHC expression with preserved interferon and antigen-processing machinery	Not established as an ecDNA-specific therapy; systemic toxicity and irreversible pathway defects may limit efficacy
Checkpoint blockade combined with ecDNA-stress therapy	Proposed DNA damage or antigen release could support immune activation	Level D for ecDNA-directed combination; conceptual or early preclinical	PD-L1, T-cell infiltration, antigen-presentation status, interferon competence, and confirmed ecDNA	ecDNA is not a validated predictor of checkpoint-inhibitor response
Antibody, antibody–drug conjugate, bispecific, or cellular therapy against an ecDNA-encoded surface target	A surface oncogene carried on ecDNA may be targetable when protein expression is clinically validated.	Target- and indication-dependent; Level C for ecDNA-specific relevance	Clinically validated surface-protein expression rather than ecDNA status alone	Current actionability derives from the expressed target; ecDNA heterogeneity may contribute to variable or reversible expression
Tumor-informed breakpoint monitoring	Patient-specific ecDNA junctions can be detected in tissue and potentially monitored in circulating tumor DNA	Level B–C; translational development	Breakpoint-resolved tumor structure followed by digital PCR or targeted sequencing	Detection of a plasma junction does not prove that an intact ecDNA circle is circulating; clinical utility remains unvalidated.
ecDNA-integrated treatment stratification	Genomic, transcriptomic, single-cell, and immune data can be integrated in research workflows.	Level D as a treatment-selection model	Whole-genome sequencing, orthogonal ecDNA confirmation, cargo, heterogeneity, replication stress, and immune context	Appropriate for biomarker research and clinical trials, not for routine treatment assignment

Evidence levels: Level A, direct ecDNA-specific evidence supported by structural confirmation and functional or longitudinal analysis; Level B, ecDNA-associated evidence from validated experimental models or clinical cohorts without complete causal isolation; Level C, evidence derived mainly from oncogene amplification, replication stress, genomic instability, or broader cancer biology; Level D, conceptual or insufficiently tested ecDNA-specific hypothesis.

ATR, ataxia telangiectasia and Rad3-related protein; BET, bromodomain and extraterminal domain; BRD4, bromodomain-containing protein 4; CDK, cyclin-dependent kinase; cGAS, cyclic GMP–AMP synthase; CHK1, checkpoint kinase 1; ecDNA, extrachromosomal DNA; EGFR, epidermal growth factor receptor; ERBB2, erb-b2 receptor tyrosine kinase 2; FGFR2, fibroblast growth factor receptor 2; FISH, fluorescence in situ hybridization; HPV, human papillomavirus; MHC, major histocompatibility complex; PD-L1, programmed death-ligand 1; RPA, replication protein A; STING, stimulator of interferon genes; WEE1, WEE1 G2 checkpoint kinase.

The strategies summarized here are evidence-ranked research approaches, not clinical recommendations. No therapy is currently approved specifically because a tumor is ecDNA-positive. When a drug targets an oncogene carried on ecDNA, treatment eligibility should follow the validated biomarker and tumor indication, not ecDNA topology alone. In current practice, validated oncogenic drivers remain actionable under tumor-specific guidelines, regardless of whether they reside on ecDNA. ecDNA status, ecDNA burden, hub architecture, breakpoint junctions, replication-stress signatures, and immune phenotypes remain candidate research biomarkers until analytical validity, clinical validity, and clinical utility are demonstrated prospectively (78–80) (**Figure 5**).

**Figure 5:**
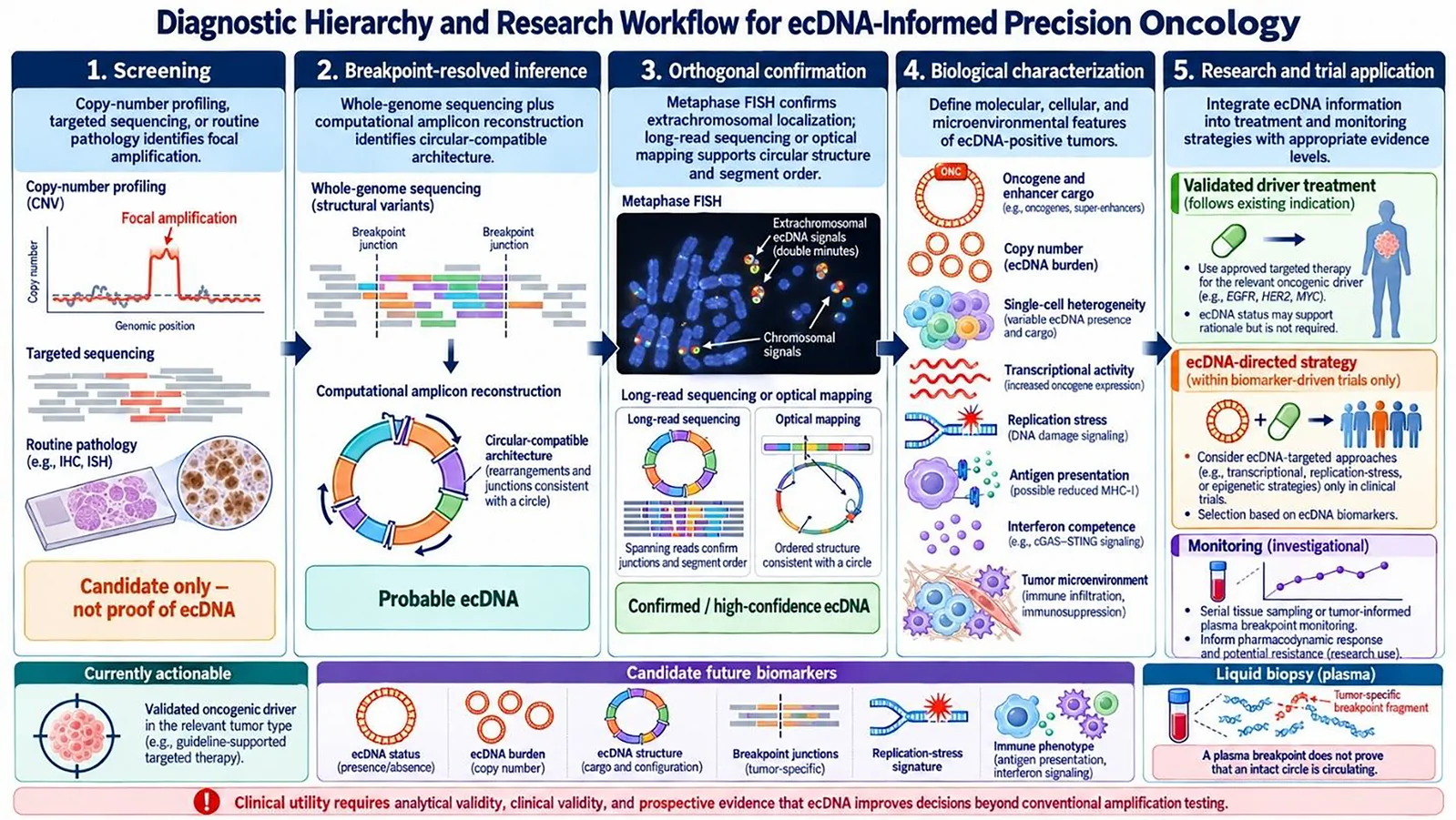
Diagnostic hierarchy and research workflow for ecDNA-informed precision oncology. Focal amplification detected by copy-number profiling, targeted sequencing, immunohistochemistry, or *in situ* hybridization identifies a candidate lesion but does not establish ecDNA. Breakpoint-resolved whole-genome sequencing and computational amplicon reconstruction can identify circular-compatible structures and support classification as probable ecDNA. Metaphase fluorescence *in situ* hybridization provides physical evidence of extrachromosomal localization, whereas long-read sequencing and optical genome mapping can strengthen reconstruction of circular architecture, segment order, and breakpoint junctions. Confirmed ecDNA-positive tumors may then be characterized by oncogene and enhancer cargo, copy-number burden, single-cell heterogeneity, transcriptional activity, replication stress, antigen-presentation status, interferon competence, and tumor-microenvironmental features. Currently, actionable treatment decisions should follow validated oncogenic drivers and tumor-specific indications. ecDNA status, burden, structure, breakpoint junctions, replication-stress signatures, and immune phenotypes remain candidate research biomarkers. Detecting a tumor-specific breakpoint in plasma does not show that an intact ecDNA circle is circulating. Clinical implementation requires analytical validity, clinical validity, and prospective evidence that ecDNA improves decision-making beyond conventional amplification testing. Schematic prepared specifically for this review; not drawn to scale.

## 15 Research gaps and future directions

First, it remains uncertain whether ecDNA topology independently promotes immune suppression. Current associations between ecDNA positivity, reduced antigen-presentation programs, altered interferon signaling, and limited immune infiltration may reflect the extrachromosomal structure, amplified oncogene cargo, chromosomal instability, tumor lineage, or the microenvironment. Isogenic models should separate these possibilities by comparing the same oncogene at comparable copy numbers on ecDNA versus within a chromosome. Functional outcomes should include major histocompatibility complex expression, peptide presentation, cytokine production, immune-cell recruitment, and tumor-cell recognition. Second, the predictive significance of ecDNA remains unresolved. ecDNA positivity is associated with aggressive disease and adverse outcomes in several cancers, supporting a potential prognostic role (81–84). However, a prognostic association does not demonstrate that ecDNA predicts sensitivity or resistance to a particular treatment. Prospective targeted-therapy and immunotherapy studies should include ecDNA-negative comparator groups, prespecified biomarker analyses, and interaction testing to determine whether ecDNA adds predictive information beyond tumor lineage, oncogene identity, amplification level, tumor mutational burden, human leukocyte antigen status, and interferon competence.

Third, clinical investigation requires a reproducible definition of ecDNA positivity. Focal amplification alone does not prove an extrachromosomal location, and short-read sequencing may infer a circular amplicon without physically confirming that all reconstructed segments coexist on a single extrachromosomal molecule. Consensus standards should distinguish screening evidence, probable ecDNA, and orthogonally confirmed ecDNA (62, 66, 77). Reports should identify the detection method, amplified cargo, copy number, breakpoint architecture, structural heterogeneity, proportion of ecDNA-positive cells, and whether circularity and extrachromosomal localization were inferred or directly demonstrated. Fourth, proposed therapeutic dependencies must be selective for ecDNA. CHK1 inhibition and enhancement of transcription–replication conflict have direct preclinical support in selected ecDNA-positive models, but clinical efficacy and predictive specificity remain under investigation (85–87). ATR, WEE1, BET proteins, transcriptional kinases, nucleotide metabolism, STING activation, hub disruption, inheritance blockade, and CRISPR-based approaches have varying and generally lower levels of ecDNA-specific evidence. Comparative studies should match ecDNA-positive and ecDNA-negative tumors for lineage, oncogenic driver, amplification level, and replication-stress phenotype. They should also determine whether ecDNA-directed pressure eliminates ecDNA or redirects evolution toward chromosomal reintegration, alternative amplification, or ecDNA-independent resistance.

Fifth, the clinical value of longitudinal ecDNA measurement is unknown. Serial tissue analysis and tumor-informed plasma assays could potentially track changes in ecDNA copy number, structure, or breakpoint abundance during treatment. However, detection of an ecDNA-derived junction in plasma does not demonstrate that an intact circle is circulating. Candidate monitoring assays require analytical validation, predefined thresholds, comparison with conventional circulating tumor DNA measurements, and prospective demonstration that an ecDNA result improves clinical decision-making. Progress in these areas will require structural genomics, functional perturbation, single-cell analysis, spatial immune profiling, and longitudinal sampling within the same studies. The immediate priority is not to expand the number of proposed pathways, but to determine which observations are genuinely caused by ecDNA, which biomarkers provide information beyond conventional amplification testing, and which dependencies offer a clinically meaningful therapeutic window. These priorities reduce the field to four decisive questions: what is caused by ecDNA topology, what is caused by its cargo, how should ecDNA positivity be defined, and does an ecDNA measurement improve a clinical decision beyond existing biomarkers?

## 16 Conclusion

ecDNA is an established mechanism of oncogene amplification, regulatory reorganization, intratumor heterogeneity, and rapid evolutionary adaptation in several cancers. Unequal segregation, altered enhancer–promoter relationships, and structural plasticity distinguish ecDNA from conventional chromosomal amplification. These properties help explain its association with aggressive disease and adaptation to selected targeted therapies. The relationship between ecDNA and tumor immune escape is plausible but remains predominantly associative and context-dependent. Some ecDNA-positive tumors show reduced antigen-presentation programs, altered interferon signaling, and immune-cell exclusion, but these findings do not establish a universal causal effect of ecDNA topology. Amplified oncogene cargo, chromosomal instability, tumor lineage, treatment history, and the microenvironment may contribute substantially to the observed immune phenotype.

Therapeutic evidence is similarly unequal. Replication-stress enhancement and CHK1 inhibition have the strongest direct preclinical support in selected ecDNA-positive models, and CHK1 inhibition is undergoing clinical investigation. ATR, WEE1, BET, CDK7, CDK9, STING-directed treatment, hub disruption, interference with ecDNA inheritance, and CRISPR-based targeting remain indirect, preclinical, or conceptual strategies. No therapy is currently established for routine use specifically because a tumor is ecDNA-positive. Clinical translation therefore depends on standardized ecDNA definitions, orthogonal structural confirmation, ecDNA-controlled mechanistic studies, and prospective biomarker trials. Until these requirements are met, ecDNA should be considered an important prognostic and evolutionary feature rather than a universally causal immune-evasion mechanism or validated treatment-selection biomarker. This evidence hierarchy provides the manuscript’s central synthesis: established structural biology should guide mechanism, but clinical claims must remain proportional to ecDNA-specific causal and prospective evidence.
